# Identification of functional long non-coding RNAs in *C. elegans*

**DOI:** 10.1186/s12915-019-0635-7

**Published:** 2019-02-18

**Authors:** Alper Akay, David Jordan, Isabela Cunha Navarro, Tomasz Wrzesinski, Chris P. Ponting, Eric A. Miska, Wilfried Haerty

**Affiliations:** 1 Wellcome CRUK Gurdon Institute, University of Cambridge, Tennis Court Road, Cambridge, CB2 1QN UK; 2 Department of Genetics, University of Cambridge, Downing Street, Cambridge, CB2 3EH UK; 3 Wellcome Sanger Institute, Wellcome Genome Campus, Hinxton, CB10 1SA UK; 4 Earlham Institute, Norwich Research Park, Norwich, UK; 5 MRC Human Genetics Unit, Institute of Genetics and Molecular Medicine, University of Edinburgh, Edinburgh, UK

**Keywords:** *C. elegans*, lncRNA, lincRNA, CRISPR, Non-coding, Long non-coding RNA

## Abstract

**Background:**

Functional characterisation of the compact genome of the model organism *Caenorhabditis elegans* remains incomplete despite its sequencing 20 years ago. The last decade of research has seen a tremendous increase in the number of non-coding RNAs identified in various organisms. While we have mechanistic understandings of small non-coding RNA pathways, long non-coding RNAs represent a diverse class of active transcripts whose function remains less well characterised.

**Results:**

By analysing hundreds of published transcriptome datasets, we annotated 3392 potential lncRNAs including 143 multi-exonic loci that showed increased nucleotide conservation and GC content relative to other non-coding regions. Using CRISPR/Cas9 genome editing, we generated deletion mutants for ten long non-coding RNA loci. Using automated microscopy for in-depth phenotyping, we show that six of the long non-coding RNA loci are required for normal development and fertility. Using RNA interference-mediated gene knock-down, we provide evidence that for two of the long non-coding RNA loci, the observed phenotypes are dependent on the corresponding RNA transcripts.

**Conclusions:**

Our results highlight that a large section of the non-coding regions of the *C. elegans* genome remains unexplored. Based on our in vivo analysis of a selection of high-confidence lncRNA loci, we expect that a significant proportion of these high-confidence regions is likely to have a biological function at either the genomic or the transcript level.

**Electronic supplementary material:**

The online version of this article (10.1186/s12915-019-0635-7) contains supplementary material, which is available to authorized users.

## Background

Transcription is not limited to the protein-coding regions of eukaryotic genomes, but instead has been observed to be pervasive in all organisms that have been studied so far. As a consequence of transcriptional activity over non-coding sections of the genomes, tens of thousands of short, < 200 nucleotides (nt) and long (> 200 nt) non-coding RNAs have now been annotated [1, 2]. While much is known about the biological role of most classes of small non-coding RNAs (e.g. microRNA, Piwi-associated RNA, small nucleolar RNA, small interfering RNA) [3–5], relatively little is known about long non-coding RNAs (lncRNAs). Whether most eukaryotic lncRNAs are functional has long been debated because of their low expression levels and rapid evolutionary turnover when compared to protein-coding genes [6, 7]. However, the molecular activities of more than a hundred of such loci have now been described [8–12] including many that appear to regulate the expression of protein-coding genes. Only a small proportion of these loci have been demonstrated to be fundamental to eukaryote biology from mutations that affect their expression or function leading to severe developmental defects or to lethal phenotypes (for example, [13, 14]). While transcription of some lncRNAs has been shown to originate at promoter or enhancer elements with potential DNA-dependent function [15], the activity of others depends on the RNA transcript, acting either in *cis* or *trans*, e.g. targeting protein complexes to chromatin or directly interacting with other RNAs, including mRNAs, lncRNAs, or microRNAs [16]. The proportions of lncRNAs belonging to each functional class remain unknown owing to painstaking experimental validations, including both knockout and knock-down assays being required.

*C. elegans* has been invaluable for the discovery of multiple non-coding RNA pathways and is an important model organism for genetic studies. Nevertheless, only one study has yet sought to identify and annotate lncRNAs in *C. elegans*, resulting in 1145 annotated loci, of which only 170 had evidence of polyadenylation [17]. Furthermore, experimental characterisation of *C. elegans* lncRNAs has been limited [18–20] and only recently a comprehensive genetic analysis of these lncRNAs have been conducted which identified 23 physiologically functional lncRNAs [21].

Using publicly available RNA-Seq libraries representing diverse *C. elegans* developmental stages, we sought to expand the annotated novel expressed long non-coding loci and to characterise informative features such as nucleotide composition, evolutionary conservation, transcript expression and functional enrichment. To assess the physiological impact of mutations within these novel lncRNAs and thus the biological importance of these loci, we used CRISPR/Cas9 to generate large genomic deletions for ten lncRNA loci. Six of these intergenic lncRNA loci yielded significant phenotypes upon deletion, and at least two of these have physiological functions that are RNA-dependent. Our study and associated experimental validation demonstrate that physiological lncRNA function in nematodes can be RNA- and/or transcription-dependent. Furthermore, we extrapolate that a significant proportion of the newly identified multi-exonic non-coding loci in the *C. elegans* genome might be functional at the genomic or the transcript level.

## Results

### Long non-coding RNA annotation in *C. elegans*

We investigated 209 publicly available RNA-Seq datasets from diverse developmental stages (Additional file 1) to annotate de novo non-coding transcripts in *C. elegans*. We decided to focus on purely intergenic loci that lack any overlap with previous coding and non-coding gene annotations. After filtering for size, coding potential and overlap with existing genes (including the previously annotated lncRNAs, see the “Methods” section), we identified 3392 long (> 200 nt) non-coding RNAs expressed across *C. elegans* development (Additional file 2). Of these 3392 lncRNAs, 143 were multi-exonic and 3249 were mono-exonic. Previously, 1145 potential lncRNAs were identified in *C. elegans* [17]. Six hundred ninety-five of these were masked in our analysis due to either being individually annotated or overlapping existing annotations. Only 18 multi-exonic and 179 mono-exonic lncRNAs identified in our analysis overlap with previous lncRNA annotations in *C. elegans*.

**Fig. 1:**
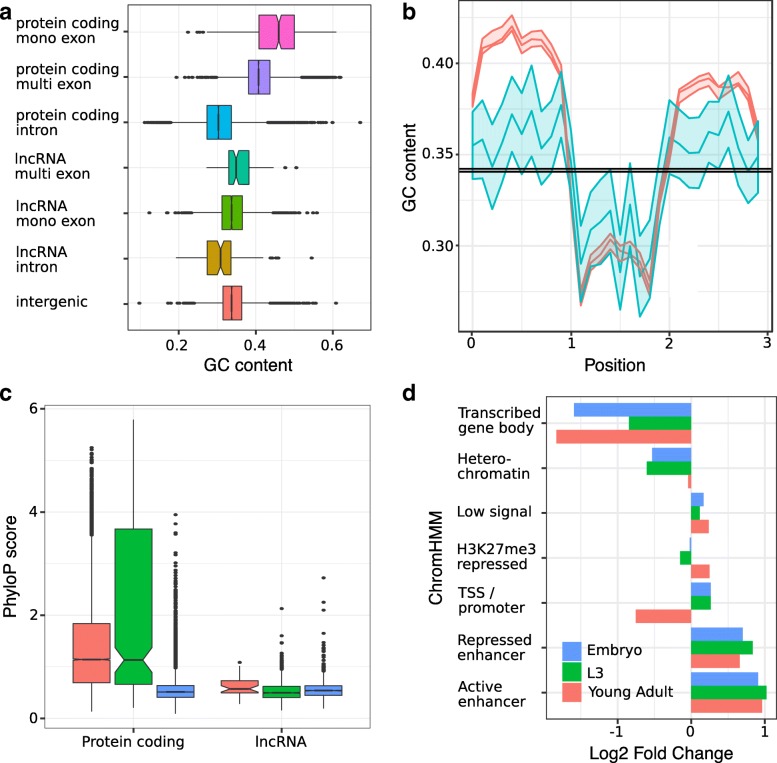
LncRNAs sequence features in *C. elegans*. **a** Nucleotide compositions of exons and introns of lncRNAs and protein-coding genes classified according to their gene model. **b** GC content variation across metagenes. The *x*-axis represents non-overlapping windows each including 10% of the sequences across multi-exonic protein-coding and lncRNA loci; the solid lines represent respectively the 95% confidence interval, median and 5% confidence interval. The black band represents the GC content of flanking intergenic sequences. **c** Nucleotide conservation (PhyloP score) comparison among intergenic sequences, multi- or mono-exonic lncRNAs and protein-coding loci. **d** Enrichment of lncRNAs for chromatin annotations identified by Daugherty et al. [28]. Transcribed gene body: ensemble of ChromHMM states characterised by H3K79me2, H3K36me3, H3K4me1 and H4K20me1. Repressed enhancers: ensemble of ChromHMM states characterised by H3K4me1 and H3K27me3. Low signal: regions without histone modification signals

CAGE data [22] was then used to accurately annotate the transcriptional orientation of 707 mono-exonic loci if found within 100 nt of the CAGE peak summit, and ChIP-Seq [23] and CLIP-Seq [24] data were used to identify transcription factor and AGO binding sites within the loci (Additional file 3). As observed in all other model organisms, the identified lncRNA loci are smaller than annotated protein-coding genes and are expressed at significantly lower levels (Additional file 4). LncRNA exons also tend to have a GC content that is lower than protein-coding sequences but higher than intronic sequences (Fig. 1a, b) as observed previously for other eukaryotes [25]. Inter-species sequence conservation for multi-exonic lncRNAs was lower than for protein-coding genes but higher than for mono-exonic lncRNAs (Kruskal Wallis test, *P* = 5.73 × 10^−5^, Fig. 1c). Our newly annotated multi-exonic lncRNAs show sequence features similar to the final set of 170 lncRNA reported by Nam and Bartel [17], (Kruskal Wallis test, *P* = 0.79 and *P* = 0.15 for nucleotide conservation and composition, respectively) showing the complementarity of these lncRNA annotations.

Distinct chromatin states inferred from histone modifications using ChromHMM [26] have been shown to associate with specific genomic elements (for example, transcriptional start sites and promoters, transcriptional elongation and gene bodies, enhancers, transposable element-derived sequences). Using previously published chromatin annotations in *C. elegans* [27, 28], we assessed the functional enrichment of our newly annotated lncRNA at each of these annotated genomic elements. Enhancers, identified either by Evans et al. [27] (1.7 fold enrichment *P* < 0.0001) or Daugherty et al. [28] (2.0 fold enrichment, *P* < 0.0001), significantly overlapped with these lncRNA loci, but chromatin states associated with transcription elongation (“transcribed gene body”) were depleted at all developmental stages (Fig. 1d, Additional file 5). These results could be explained by the observed low expression level of the lncRNAs. Our results are in agreement with Evans et al. [27], who showed that the chromatin states reflecting transcription elongation were associated with the most highly expressed genes in their study. Active enhancers were particularly enriched within single exon lncRNAs at all developmental stages (2.0, 2.6 and 2.4 fold enrichment at the early embryonic, L3 and young adult stages, respectively, *P* < 0.001 in all comparisons, Additional file 5). This result is consistent with enhancer RNAs rarely being spliced [29]. In contrast, multi-exonic loci were only enriched for active enhancers during early embryonic stage (2.3 fold enrichment, *P* < 0.0001) which likely reflects the fewer number of multi-exonic lncRNA expressed at later stages.

**Fig. 2:**
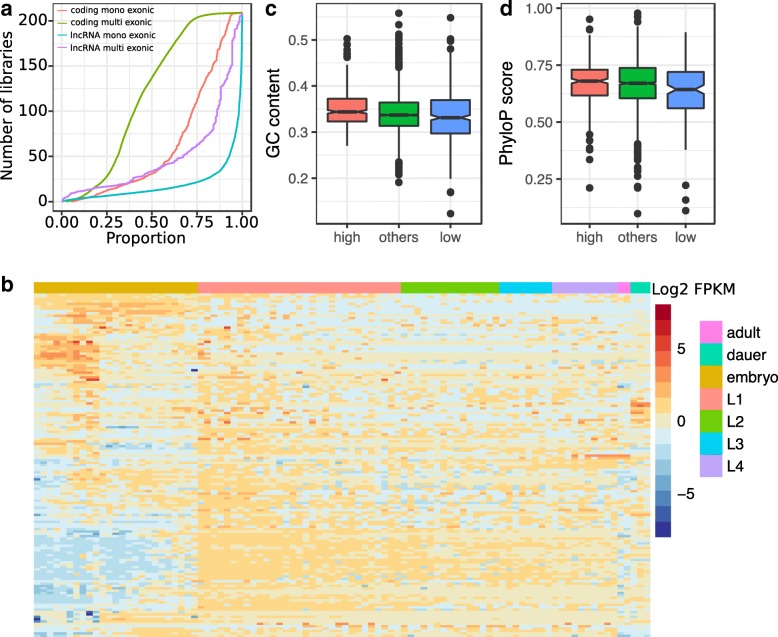
**a** LncRNA expression properties. **a** Cumulative distribution of the proportion of multi-exonic, mono-exonic lncRNA and protein-coding loci identified as expressed across all libraries (FPKM > 1). **b** Expression (log_2_ FPKM) across *C. elegans* development of 143 multi-exonic lncRNAs. Each column represents the average expression at one time point for whole individuals in standard conditions. GC composition (**c**) and nucleotide conservation (**d**) for lncRNA loci depending on the reproducibility of lncRNA model predictions across libraries. ≤ 20 of the libraries (low), 20 to 100 (others) and ≥ 100 libraries (high)

Half of all lncRNA loci are expressed in at least 12 libraries (FPKM > 1; or at least 41 libraries if FPKM > 0.1; Fig. 2a). This restricted expression could reflect that many of the newly annotated loci are the result of transcriptional noise and therefore likely non-functional [15]. However, many of the remaining lncRNAs, most specifically multi-exonic lncRNAs, appear to be expressed in a tissue- and stage-specific manner (Fig. 2b). Twenty-six lncRNAs (10 multi-exonic) were expressed in more than 90% of the libraries (≥ 188 libraries). Highly reproducible loci (≥ 100 libraries) tended to have a significantly higher sequence conservation (Kruskal Wallis test, corrected *P* = 0.0077) and higher GC content (Kruskal Wallis test, *P* = 3.5 × 10^−4^ after Bonferroni correction) compared with loci with limited reproducibility (< 10% libraries) (Fig. 2c, d). Highly reproducibly expressed loci also tended to have stronger enrichment for enhancer regions identified in embryos (2.2–3.1 fold enrichment) or in L3 larvae (2.7–4.4 fold enrichment, Additional file 5).

### Functional characterisation of lncRNA loci

**Fig. 3:**
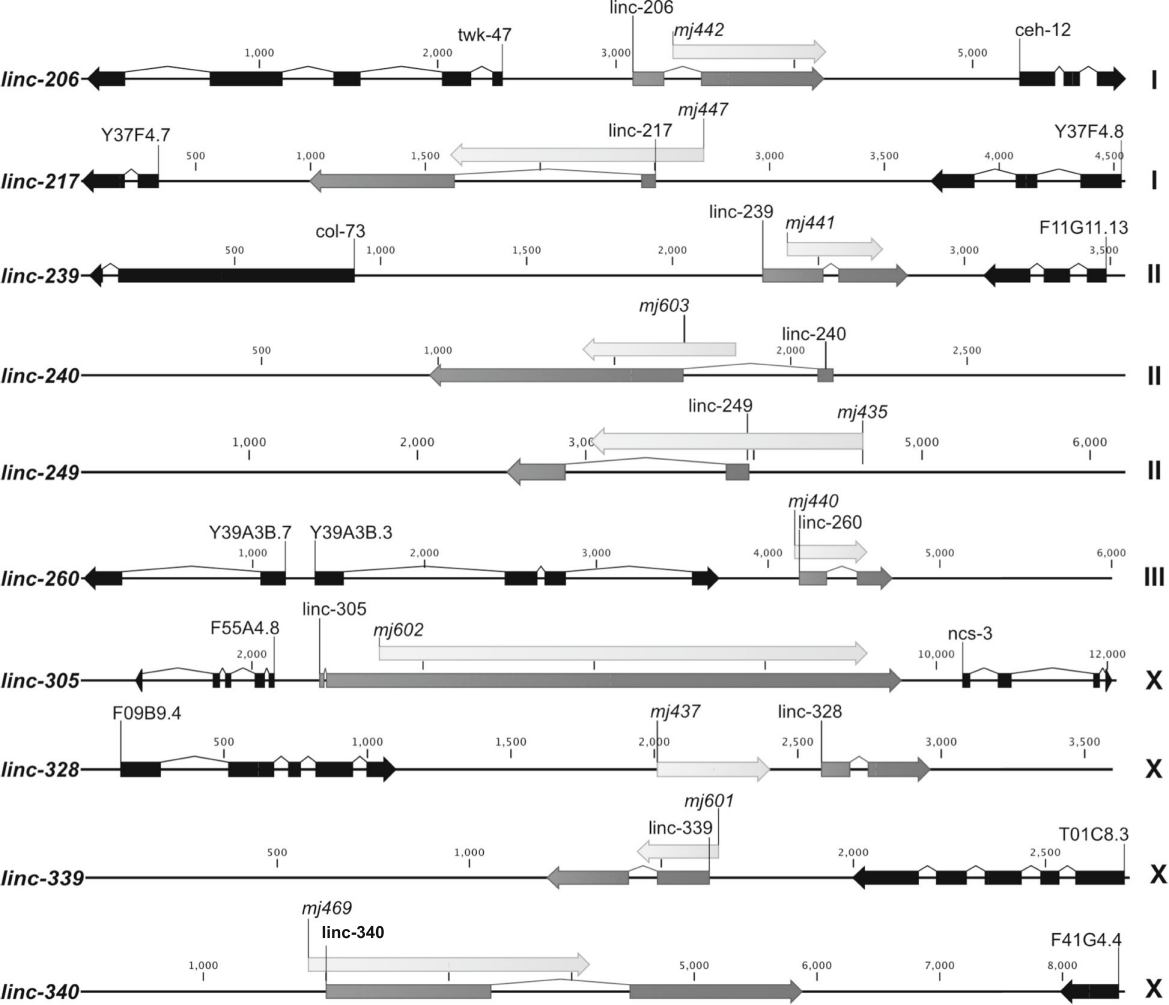
Gene structure of the ten lncRNA deletions. lncRNAs (dark grey), neighbouring protein-coding genes (black) and deleted regions (light grey and named with the respective allele name, *mj*) are shown. Numbers are to an arbitrary start point and do not reflect chromosomal location. Chromosomes are indicated at the right hand side

Higher sequence conservation of the 143 multi-exonic lncRNA loci, together with their higher exonic GC content and their splicing, could reflect organismal function. To test this hypothesis, we used CRISPR/Cas9 genome editing to generate targeted deletions in ten of the multi-exonic lncRNA loci that each showed high sequence conservation, high expression and evidence from multiple libraries and that are not overlapping with neighbouring coding regions. We were successful in generating large genomic deletions for ten of these lncRNA loci (Table 1) out of 20 that were initially targeted. This success rate was due mostly to the limited efficiency of plasmid-based CRISPR/Cas9 genome editing protocols that were used early in the study, as compared to direct protein/RNA injection methods [30]. Of these lncRNA locus deletions, nine removed at least one exonic region and one removed a region just 5′ of a lncRNA locus (Fig. 3).

**Table 1:** List of lncRNAs deletions and their phenotypes

Strain	lncRNA	Locus	Chr	RNAi clone #	Reduced viable progeny (mutant)	Smaller body size (deletion mutant)	Reduced viable progeny (RNAi)	Smaller body size (RNAi)
SX3268	linc-206*	XLOC_000670	I	112	–	+	–	–
SX3278	linc-217*	XLOC_003573	I	111	+	+	–	–
SX3269	linc-239*	XLOC_005681	II	106	+	+	+	+
SX3332	linc-240	XLOC_008459	II		–	–		
SX3340	linc-249	XLOC_009275	II	108	+	+	–	–
SX3270	linc-260	XLOC_010885	III		+	–		
SX3313	linc-305	XLOC_040158	X		+	–		
SX3338	linc-328	XLOC_041869	X		–	–		
SX3315	linc-339	XLOC_045957	X	107	+	–	+	+
SX3271	linc-340	XLOC_047005	X	109	–	–	–	–

*lncRNAs that overlap recent predicted protein-coding genes

**Fig. 4:**
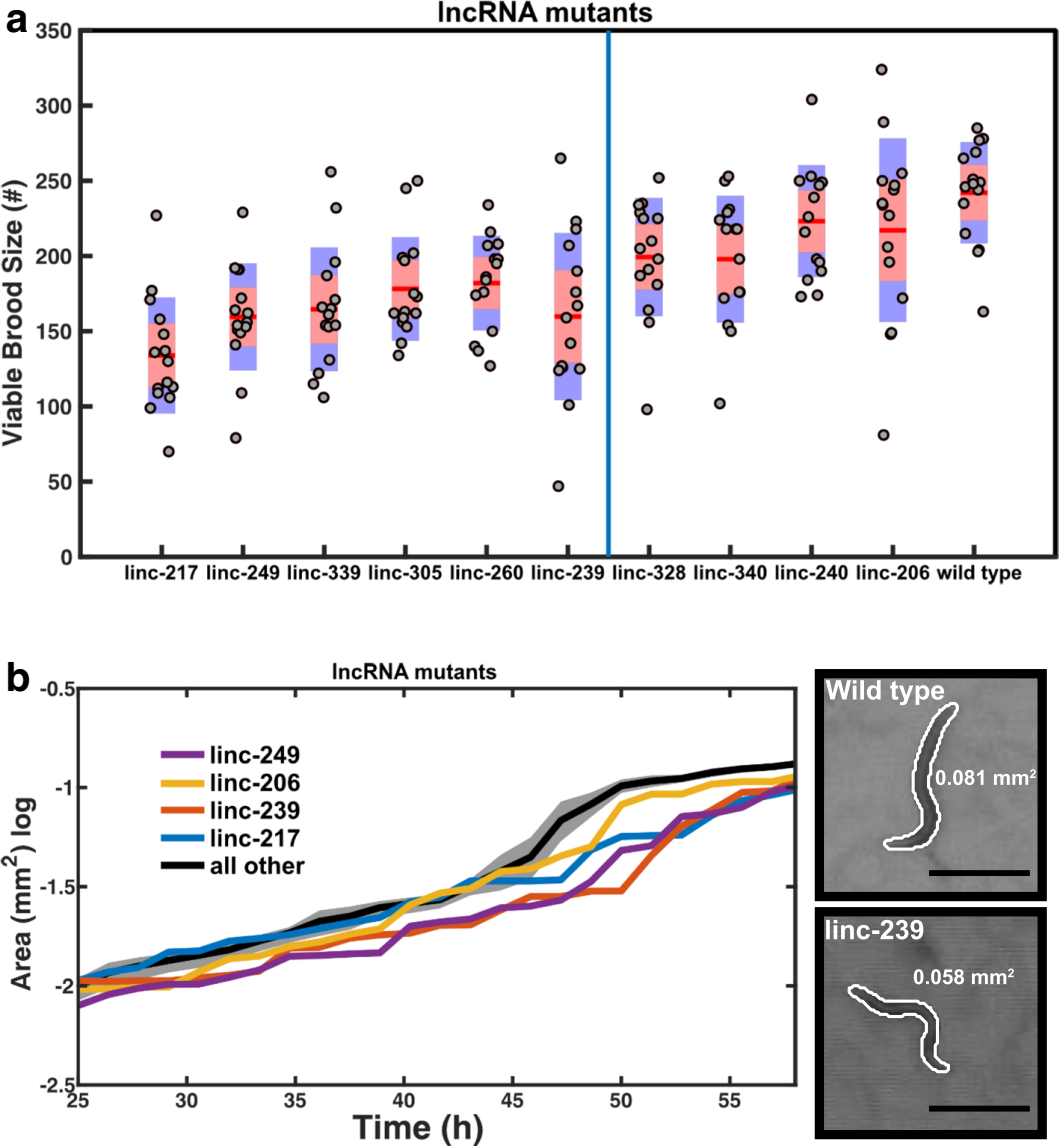
Phenotyping of ten lncRNA deletion mutants. **a** Viable brood sizes are presented with their standard deviations (blue area) and the 95% confidence interval of the mean (red area). Samples were compared to wild type animals using a pairwise two-sample *t* test with a multiple test (Bonferroni) correction. Samples are ordered by increasing *p* value and those found to be significant at (*p* ≤ 0.05) are shown to the left of the blue line (*n* = 15 animals/mutant). **b** Growth curves were compared to wild type animals, and those found not to be significantly different are shown by their mean across strains (black line) with the standard error of the mean (grey area). Those found to be significantly different from the control are shown individually as means only. Inset shows example images of the wild type (top) and linc-239 mutant (bottom) at 45 h post hatching with the computer-generated outlines, and computed area (black line = 500 μm)

All ten lncRNA deletion mutants initially failed to display overt, gross phenotypes such as sterility, embryonic lethality, larval arrest or abnormal body development. To undertake a more extensive characterisation, we captured the development of the mutant animals alongside wild type control animals using an automated microscopy system. This system records the development of multiple animals simultaneously and permits phenotypic analysis in an unbiased manner. Two phenotypes that can influence the life history and fitness of populations [31], brood size and growth rate, were selected for the automated analysis. Six of ten lncRNA deletion mutants (linc-217, linc-239, linc-249, linc260, linc-305 and linc-339) yielded significant reduction in viable brood size (Fig. 4a) and four of 10 mutants (linc-206, linc-217, linc-239 and linc-249) displayed reduced growth rate (slower body size increase) over development (Fig. 4b). Three mutants (linc-217, linc-239 and linc-249) showed alterations of both phenotypes (Table 1, Fig. 4a, b).

These phenotypes could be due to the removal of either the lncRNA transcript or of the genomic locus which, in some instances, harboured annotated transcription factor binding and enhancer sites (Additional file 3). To distinguish between these two possibilities, we generated dsRNA expression vectors for RNAi targeting of the lncRNA transcripts in wild type animals (Table 1, Additional file 6). Using four biological replicates per assay, we targeted six lncRNA transcripts using RNAi. We left out linc-240, linc-328, linc-260 and linc-305 because these were either lacking any phenotype or yielded only a weak phenotype when partially or fully deleted.

**Fig. 5:**
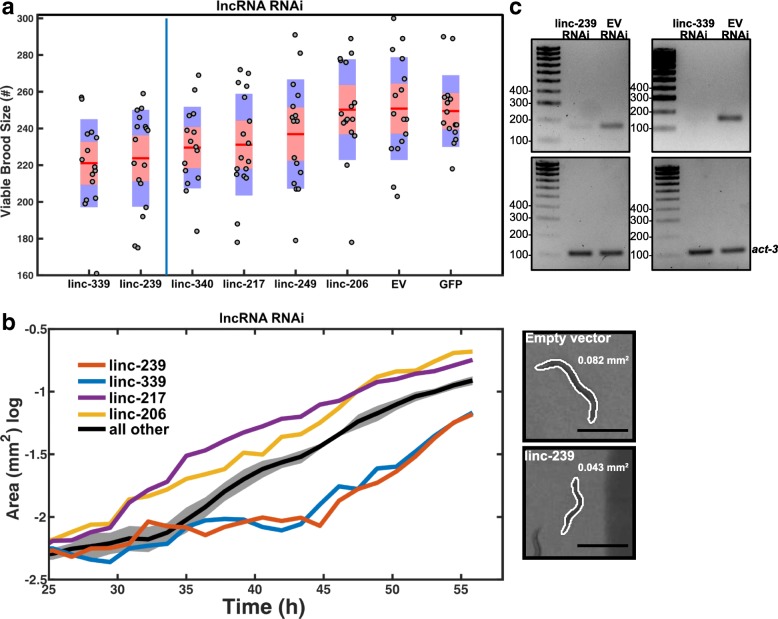
RNAi-mediated knock-down of lncRNAs. **a** Viable brood sizes are presented with their standard deviations (blue area) and the 95% confidence interval of the mean (red area). Samples were compared to “empty vector” control animals using a pairwise two-sample *t* test with a multiple test (Bonferroni) correction. Samples are ordered by increasing *p* value, and those found to be significant at (*p* ≤ 0.05) are shown to the left of the blue line (*n* = 18 animals/mutant). Empty vector (EV), GFP and linc-340 RNAi are negative controls. **b** Growth curves were compared to “empty vector” animals, and those found to be not significantly different are shown by their mean across strains (black line) with the standard error of the mean (grey area). Those found to be significantly different from the control are shown individually as means only. Inset shows example images of the “empty vector” (top) and linc-239 RNAi (bottom) at 45 h post hatching with the computer-generated outlines, and computed area (black line = 500 μm). **c** RT-PCR analysis of RNAi knock-down efficiency for linc-239 and linc-339. Actin is used as loading control

**Fig. 6:**
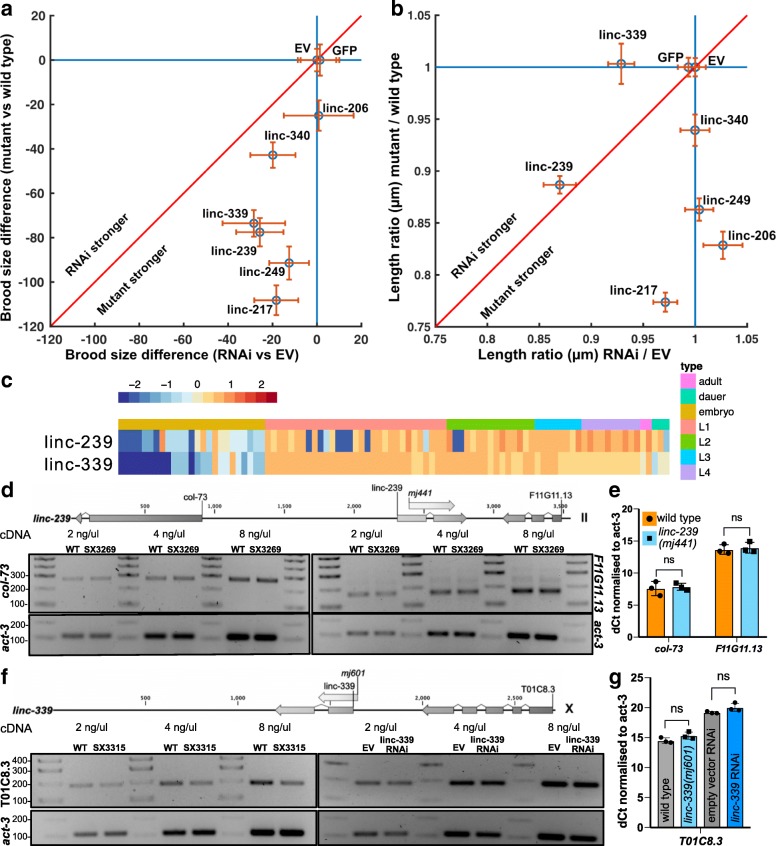
Comparison of phenotypes arising from lncRNA genomic deletion mutants and phenotypes arising from RNAi-mediated knock-down of lncRNA transcript. To compare the effects of the disruption of a lncRNA genomic locus to the knock-down of the corresponding lncRNA transcript by RNAi, the mean brood size reduction compared to the control (**a**) and the ratio of the length at 50 h relative to the control (**b**) were plotted. These are shown as a scatter plot of the mean reduction (**a**, blue circle) or the mean ratio (**b**, blue circle) with the 95% confidence interval of the mean (orange lines). If the mutations or the RNAi yield an effect, data fall below the line *y* = 1 and to the left of *x* = 1. If mutants and RNAi yield similar effects, data fall along the red line; above the red line indicates that RNAi has a greater effect, while below the red line indicates that the genomic mutation has a greater effect. **c** Expression (log_2_ FPKM) across *C. elegans* development for linc-239 and linc-339. **d** RT-PCR analysis of neighbouring genes in linc-239 *(mj441)* deletion mutant. Diagram shows the relative position of the lncRNA and the surrounding protein-coding genes. *Col-73* expression (left panel) and F11G11.1 expression (right panel) are measured using end-point RT-PCR at three different cDNA concentrations. Actin is used as loading control. **e** qRT-PCR analysis of *col-73* and F11G11.13 expression in linc-239 *(mj441)* mutants. Error bars = 95% CI, significance is tested using *t* test with multiple *t* test correction. **f** RT-PCR analysis of the T01C8.3 gene in *linc-339* (*mj601*) deletion mutants (left panel) and in linc-339 RNAi knock-down (right panel) at three different cDNA concentrations. Actin is used as loading control. **g** qRT-PCR analysis of T01C8.3 expression in linc-339 (*mj339*) and linc-339 RNAi. Error bars = 95% CI, significance is tested using *t* test with multiple *t* test correction

Of the four of these six lncRNA loci whose deletion yielded a reduced viable brood size phenotype, two (linc-239 and linc-339) yielded an equivalent phenotype when expression was reduced using RNAi (Fig. 5a); for one of these lncRNA loci, linc-239, equivalent reduced growth rate phenotypes were observed for both its knockout and knock-down (Table 1, Fig. 5b). These results indicate that for these two loci, the phenotypes are caused by the disruption of their RNA transcript-dependent functions. We further validated the transcript expression and RNAi knock-down efficiency of linc-239 and linc-339 using RT-PCR and observed a strong reduction in linc-239 and linc-339 transcript levels (Fig. 5c and Additional file 7). RNAi targeting of another lncRNA, linc-339, also showed a reduced growth rate, a phenotype that was not observed in the deletion mutant (Fig. 5b). Three additional lncRNA strains (linc-206, linc-217, linc-249) yielded discordant phenotypes when disrupted or subjected to RNAi (Table 1, Figs. 4 and 5). This would be consistent with functions of these loci being RNA-independent. Direct comparison of the phenotypes between lncRNA deletion mutants and RNAi knock-down of lncRNAs shows that RNAi knock-down phenotypes are slightly weaker or equivalent to deletion mutants (Fig. 6a, b). We calculated the expression of linc-239 and linc-339 to be highest during larval development in comparison to embryogenesis (Fig. 6c). The phenotypes associated with linc-239 and linc-339 can arise from their *cis* regulatory function on the neighbouring genes. We analysed the expression levels of genes that are in close proximity to the linc-239 and linc-339. linc-239 has two neighbouring genes, *col-73* and F11G11.13. In linc-239 (*mj441*) deletion mutants, we did not observe any change in the expression levels of *col-73* and F11G11.13 using both end-point RT-PCR and qRT-PCR (Fig. 6d, e). In linc-339 (*mj601*) deletion mutants, we observed a small reduction in the expression of its neighbouring gene T01C8.3 using RT-PCR (Fig. 6f). Both the deletion of linc-339 and its RNAi knock-down lead to comparable reduction in viable brood size. If reduction in T01C8.3 expression is required for this phenotype, then linc-339 RNAi knock-down should similarly affect the T01C8.3 expression. Instead, we observed that the T01C8.3 expression does not change upon linc-339 RNAi knock-down (Fig. 6 f, right hand panel). We further tested the expression of T01C8.3 using qRT-PCR (Fig. 6g) and did not observe any significant change in expression when the linc-339 is either deleted or knocked-down. Our results suggest that both linc-239 and linc-339 are likely to function *in trans*.

## Discussion

The identification of functional non-coding elements, including transcribed non-coding sequences, in genomes has long relied on computational predictions based on sequence conservation [32], or biochemical activity [33]. However, regardless of the preferred approach to predict functional sequences, only experimental validation can truly substantiate the inferred functionality of an element.

In our study, we first provide a novel annotation of intergenic lncRNAs in *C. elegans.* This work expands on the previous annotations delivered by Nam and Bartel [17] as a substantially more comprehensive RNA-Seq dataset was available at the time of our study (209 vs 35). We also took advantage of existing resources to further improve the annotations for these loci. These included not only their expression pattern and nucleotide conservation but also (i) the presence of potential functional elements within them (transcription factor binding and AGO binding sites), (ii) their correlation in expression with neighbouring protein-coding genes and (iii) the reported mutant phenotypes for these genes. The primary aim of these comprehensive annotations was to inform the selection of candidate lncRNA loci for follow-up experimental validation. Most importantly, we went beyond computational predictions of functionality as we assessed the in vivo phenotypic effect of knocking-out a selection of ten intergenic lncRNAs using automated microscopy, and implemented knock-down assays to validate the observed brood size and growth rate reduction and putative transcript-mediated function of these loci.

### lncRNAs of *C. elegans*

Our newly annotated loci bear all the hallmarks of lncRNAs in other organisms: they tend to be shorter, expressed at lower levels and have a lower degree of conservation than protein-coding sequences [6, 7, 11]. Furthermore, the GC composition of the multi-exonic lncRNAs in *C. elegans* does mirror the patterns previously observed in other animals, with increased GC content within exons relative to introns [25]. These similarities with other animal lncRNA annotations implicate *C. elegans* as a model organism that is more broadly relevant for investigation of the molecular functions of lncRNAs and the processes through which those functions are conveyed. Most importantly, the wealth of resources available for *C. elegans* as a model organism offer the opportunity to assess the in vivo impact of mutations within these loci.

The observation of enrichment for enhancer sequences within our lncRNA loci emphasises that the observed function of a locus could be conveyed by discrete functional DNA elements located within it rather than by the RNA transcribed at this location. The former would imply that transcription at this location either reflects or maintains open chromatin states and that the resulting transcript would likely be biologically inconsequential, whereas the latter would imply RNA sequence-dependent functionality of the resulting transcript [10, 34]. LncRNAs with transcript-mediated function have been shown to act both *in cis* (Xist [35]) and *in trans* (Paupar [36]), whereas those whose function is transcription regulation related are expected to act in *cis* (transcriptional interference, chromatin modification at enhancers). This duality, transcription- versus transcript-mediated function, is a recurrent issue when studying lncRNAs and only the careful experimental characterisation of each locus through knockdown, and rescue can begin to deduce the functional mechanism associated with a non-coding transcript [37].

### Phenotypic characterisation of lncRNAs

Historically, majority of *C. elegans* genes were identified through genetic screens which concurrently provide phenotypic and functional information. Mutations identified in non-coding regions of the genome as a result of genetic screens, nevertheless, have largely remained uncatalogued. With advances in genome editing methods, it is now possible to directly target non-coding regions for mutational analysis. The lncRNA annotations presented in this study, together with the detailed documentation of their expression and overlap with existing datasets, serve as a guide for the targeted analysis of these loci during animal development.

In *C. elegans*, many small non-coding RNA genes lack discernible phenotypes when deleted individually [38]. This is mostly due to redundancy between non-coding RNAs and the role such RNAs play as buffers in gene expression regulation rather than being the master regulators. The situation may be similar for the majority of lncRNAs, because their roles in the regulation of gene expression remain incompletely understood and the phenotypic characterisation of many vertebrate lncRNAs has been challenging and has provided sometimes contradictory results [39]. The genomic deletions of the ten lncRNA sequences described in this study failed to display any gross phenotype. This is in line with another study published during the revision of our work which described a comprehensive deletion analysis of the previously annotated lncRNAs in *C. elegans* [21]. By using automated microscopy, we sought to capture the phenotypes associated with lncRNAs in an unbiased manner. While this approach gives greater power to screen larger number of individuals and accurately quantify phenotypes, more subtle phenotypes or phenotypes more difficult to quantify might not be captured. The observed reductions in brood size and growth rate of lncRNA loci deletion mutants greatly affect the fitness of these animals, despite their otherwise normal appearance. For two of the lncRNA loci, linc-239 and linc-339, the phenotypes can be recapitulated by RNAi knock-down. We thus consider these two lncRNAs as being representative of bona fide *C. elegans* lncRNAs. However, further experiments will be required to completely rule out that these lncRNAs are not translated into functional, short polypeptides [40]. It is also important to note that according to the most recent genome annotations (WS269), both linc-206 and linc-217 overlap protein-coding sequences. The same issue also exists with linc-239 which appears to partially overlap a recently annotated single exon coding transcript (F11G11.15) which is supported only by weak evidence and marked as potentially non-coding in Wormbase. The lack of phenotypes upon RNAi knock-down of the remaining loci could be attributed to the possibility that observed phenotypes in deletion mutants arising from the removal of DNA-dependent functional elements. It is also possible that the transcripts of these loci are solely nuclear or expressed in neuronal tissues and thus resistant to RNAi in *C. elegans* [41, 42].

## Conclusions

In this study, we increased the current number of potential lncRNAs in *C. elegans* from 801 to 4001. Together with the previously identified high-confidence lncRNA loci, in total 298 loci yield evidence for possible biological functions because they display higher conservation, higher expression, higher GC content and splicing. Using genome editing and RNA interference methods, we tested the functional relevance of ten of these loci and demonstrated that six yield in vivo phenotypes when deleted. Furthermore, we showed that for at least two out of these six loci, the function is likely conveyed by the RNA transcript. From our in vivo assays, we estimate by extrapolation that 40–60% of the multi-exonic lncRNAs identified in this study might have biological roles. It will be essential to employ sensitive experimental approaches to decipher the fitness effect of such non-coding loci.

## Methods

### Intergenic lncRNA identification

A total of 209 publicly available libraries were retrieved from the SRA database (https://www.ncbi.nlm.nih.gov/sra/, Additional file 1). Reads were mapped onto the *C. elegans* (ENSEMBL release 73, WBcel235) reference genome using TOPHAT2 (2.0.9) [43] with default parameters. For each library, de novo transcripts were called using cufflinks2 (2.2.1) [44] and the coding potential of all new intergenic transcripts was assessed using the Coding Potential Calculator (CPC, 0.9-r2 [45], score < 0) as well as CPAT (1.2.4 [46], score < 0.403). All of the loci for which every transcript was deemed non-coding were retained for further analyses as potential intergenic lncRNAs. All lncRNAs across all libraries were merged into a single annotation file using Cuffcompare. We retained for final analyses only the loci longer than 200 nt, not overlapping any annotated gene and found at least 50 nucleotides away from annotated genes if located on the same strand.

We downloaded the Nam and Bartel lncRNAs [17] for further comparison with our data set. Out of the 1195 loci, we found that the 170 high-confidence Nam and Bartel lncRNAs are included in existing annotations and an additional 525 low-confidence loci overlap existing genes.

### Intergenic lncRNA conservation

The nucleotide conservation of the candidate loci was assessed using the conservation tracks (PhyloP) from the UCSC database (http://hgdownload.soe.ucsc.edu/). The tracks represent the nucleotide conservation across 26 nematode species.

### Chromatin modifications, transcription binding sites and enhancers associated with lncRNAs

In order to facilitate the prioritisation of lncRNAs for mutagenesis, we parsed publicly available data to further improve our annotations. We intersected our annotated loci with highly occupied target regions [47], miRNA binding sites [24], transcription factor binding sites identified by modENCODE [23] and enhancers identified by Chen et al. [22]. We also computed the distance to the closest protein-coding gene as well as the correlation in expression between lncRNAs and their upstream and downstream flanking protein-coding genes. Finally, we also reported the known phenotypes for the proteins flanking lncRNAs. Genomic locations of the respective annotations were transferred to the ce11 genome assembly using liftOver and files available on the UCSC database. Enrichment analyses were performed using the Genomic Association Test (GAT) software [48].

### Identification of transcriptional start sites

We used the 5′ end tag sequencing data from Chen et al. [22] to identify the putative transcriptional start sites of the intergenic lncRNAs. We applied the same approach the authors previously applied to their data. Clusters with at least two reads were kept and merged if on the same strand and within 25 nucleotides of each other. We used the strand information of the CAGE peaks to inform the transcriptional orientation of mono-exonic loci found within 100 nt of a CAGE peak summit.

### CRISPR/Cas9-mediated deletion of lncRNA loci

lncRNA loci were deleted using either plasmid base injection [49, 50] or direct protein/RNA injection methods [30, 51], as previously described. gRNA sequences and the primers used for screening of the F2 generation animals are given in Additional file 6. Isolated deletion mutants were backcrossed once to wild type animals. For lncRNA sequences and deletions, see Additional file 8; for genotyping results of deletion mutants, see Additional file 9.

### Cloning of RNAi vectors

Genomic sequences corresponding to the lncRNA loci (Additional file 6) were cloned using Gibson Assembly [52] into the L4440 vector and transformed into competent *E. coli* strain HT115 [41].

### RT-PCR and qRT-PCR analysis of lncRNA expression

Total RNA was isolated using TriSure reagent and chloroform extraction, followed by isopropanol precipitation. Genomic DNA was removed using TurboDNA Free kit (Thermo) according to manufacturer’s recommendations. cDNA synthesis was done using SuperScript II reverse transcriptase according to manufacturer’s instructions starting from 0.5–1 μg RNA. cDNA was directly used in PCR reactions after dilution as indicated in individual figures. In Fig. 5 and Additional file 7 35 cycles of PCR amplification was used. In Fig. 6, 25 cycles of PCR amplification was used. RT-PCR primers are listed in Additional file 6. qRT-PCR was done using Applied Biosystems PowerSYBRGreen PCR master mix and an Applied Biosystem StepOnePlus machine. Delta Ct calculations were done using the Ct values of the control gene *act-3*. qRT-PCR data is generated using three biological replicates.

### Preparation of RNAi plates

*E. coli* HT115 bacteria transformed with individual RNAi expression vectors were grown in LB-ampicillin (50 μg/ml) until OD600 measurement of 0.6–0.8. Bacteria were seeded onto NGM agar plates containing a 1-mM IPTG and 25-μg/ml carbenicillin. Worms were added onto the plates as described below for growth curve and brood size measurements after at least two generations of RNAi feeding.

### Automated microscopy analysis

#### Brood size

Brood size measurements were completed over three 24-h intervals. First, eggs were prepared by synchronisation via coordinated egg-laying. When these animals had grown to the L4 stage, single animals were transferred to fresh plate (day 0). For 3 days, each day (days 1–3), each animal was transferred to a new plate, while the eggs were left on the old plate and allowed to hatch and grow for ~ 3 days, after which, the number of animals on each of these plates was counted [31] using a custom animal counting program utilising short video recordings. Animals were agitated by tapping each plate four times, after this, 15 frames were imaged at 1 Hz and the maximum projection was used as a background image. Animals were then detected by movement using the difference image between each frame and this background image and counted this way for ten additional frames. The final count was returned as the mode of these counts. This system was tested on plates with fixed numbers of animals and was accurate to within 5%, comparable to human precision. Total brood size was reported then as the sum for 3 days. For mutant strains, this experiment was done for five animals of each strain three times. For the RNAi experiment, this was done for six animals for each RNAi clone, also done three separate times. Data is censored for animals that crawled off of plates. See Additional file 10 for comparison of automated microscopy accuracy to manual counting. Raw data of brood size counts are in Additional file 11.

#### Growth curves

Growth curves were estimated using long-term video imaging. In short, a custom camera system was used to record backlit images of *C. elegans* from the ex utero egg stage to the egg-laying adult stage (~ 65 h). To accomplish this, an imaging system was built, which allowed 12 video cameras (Flea3 3.2MP monochrome, Point Grey) to record in parallel. These were used to record images of 40 *C. elegans* nematodes in 16-mm circular arenas continuously at 1 Hz for ~ 3 days. These “mini-wells” were placed in an enclosure where temperature was maintained at 20 °C ± 20 mK. The resulting movies were analysed off-line with a custom written MATLAB script (Mathworks). Tracking was based on the Hungarian Algorithm for linear assignments [53–55] and yielded spatial trajectories *r*(*n*, *t*) and time series of attributes such as the area of the 2D projection *A*(*n*, *t*) and the length along the centerline *l*(*n*, *t*), where *n* denotes the individual and *t* denotes the time. Growth curve data were calculated by first taking the time average at time *s* in a window of length *w*, ${A}_n(s)=\overline{A\left(n,s:s+w\right)}$. The population average in that window is then the ensemble average of the individual averages *A*(*s*) = 〈*A*_*n*_(*s*)〉. For these analysis, *w* was set at 20 min. Additionally, in each window, the standard error of the mean was computed. In each set of experiments, those with mutants, and those done with RNAi bacteria, a standard growth curve was selected, the *C. elegans* N2 strain and the *empty vector* (*ev*) bacteria, respectively. For visual clarity, any other growth curves that fell within the 99% confidence interval of the “standard” curve were combined *A*_*j*_ : 〈{*A*_*j*_(*s*) : *p*(*H* ≠ *H*_0_(*A*_*j*_, *A*_*std*_) < 0.01}〉. Those that did not fall into this set were plotted individually (see Additional file 12 for raw growth rate data and Additional file 13 for length data (related to RNAi)).

## Additional files

Additional file 1: List of the RNA-Seq libraries used to annotate lncRNAs (XLSX 20 kb)

Additional file 2: Annotation of the novel lncRNAs in *C. elegans* (GTF 878 kb)

Additional file 3: **a** Annotation of the lncRNAs in *C. elegans*, including genomic position, multi-exonic or mono-exonic nature, distance to CAGE peak, distance to closest locus, overlap with previously annotated lncRNA, overlap with epigenomic marks and miRNA binding sites, GC proportion, nucleotide conservation, number of libraries in which the locus is expressed, median and mean expression (FPKM), stage of highest expression, closest upstream and downstream protein-coding genes, correlation of expression and associated *P* value, reported phenotype for the protein. **b** Reported phenotypes for upstream and downstream protein-coding genes of knockout and knock-down lncRNAs (XLSX 810 kb)

Additional file 4: Comparison of protein-coding and lncRNA transcript size (PDF 66 kb)

Additional file 5: Enrichment of lncRNAs (PDF 78 kb)

Additional file 6: Primers and gRNAs used in this study. The strain name (SX), allele name for deletions (mj), lincRNA loci (XLOC), CRISPR guide RNA sequences (CRISPR gRNA), genotyping primers and RT-PCR primers are shown. Oligos are named with a prefix “M” followed by a number identifying their location in the local laboratory database. Similarly plasmids are identified by a prefix “pEM” followed by a number (XLSX 13 kb)

Additional file 7: RT-PCR of 2 lncRNAs, linc-239 and linc-339. End-point RT-PCR analysis of linc-239 and linc-339 in wild type and respective mutant animals (PDF 92 kb)

Additional file 8: Sequence of 10 lncRNAs and deletions. Full sequence information of the lincRNAs and their deletions. The legend is within the file (RTF 46 kb)

Additional file 9: Genotyping of 10 lncRNA mutants. PCR and gel electrophoresis analysis of deletion mutants for the described lincRNAs (PDF 341 kb)

Additional file 10: Extended methods for automated microscopy and phenotyping (PDF 197 kb)

Additional file 11: Raw brood size measurement data (CSV 7 kb)

Additional file 12: Raw data of growth rate measurements (CSV 10 kb)

Additional file 13: Raw data of length measurements (CSV 4 kb)
