# Supplementary material for: Identification of functional long non-coding RNAs in C. elegans
Source: BMC Biol. 2019 Feb 18;17:14. doi: 10.1186/s12915-019-0635-7 (PMC6378714; doi:10.1186/s12915-019-0635-7)

Additional File 4. Comparison of protein coding and lncRNA transcript size (longest transcript) and expression (Average expression across all libraries).

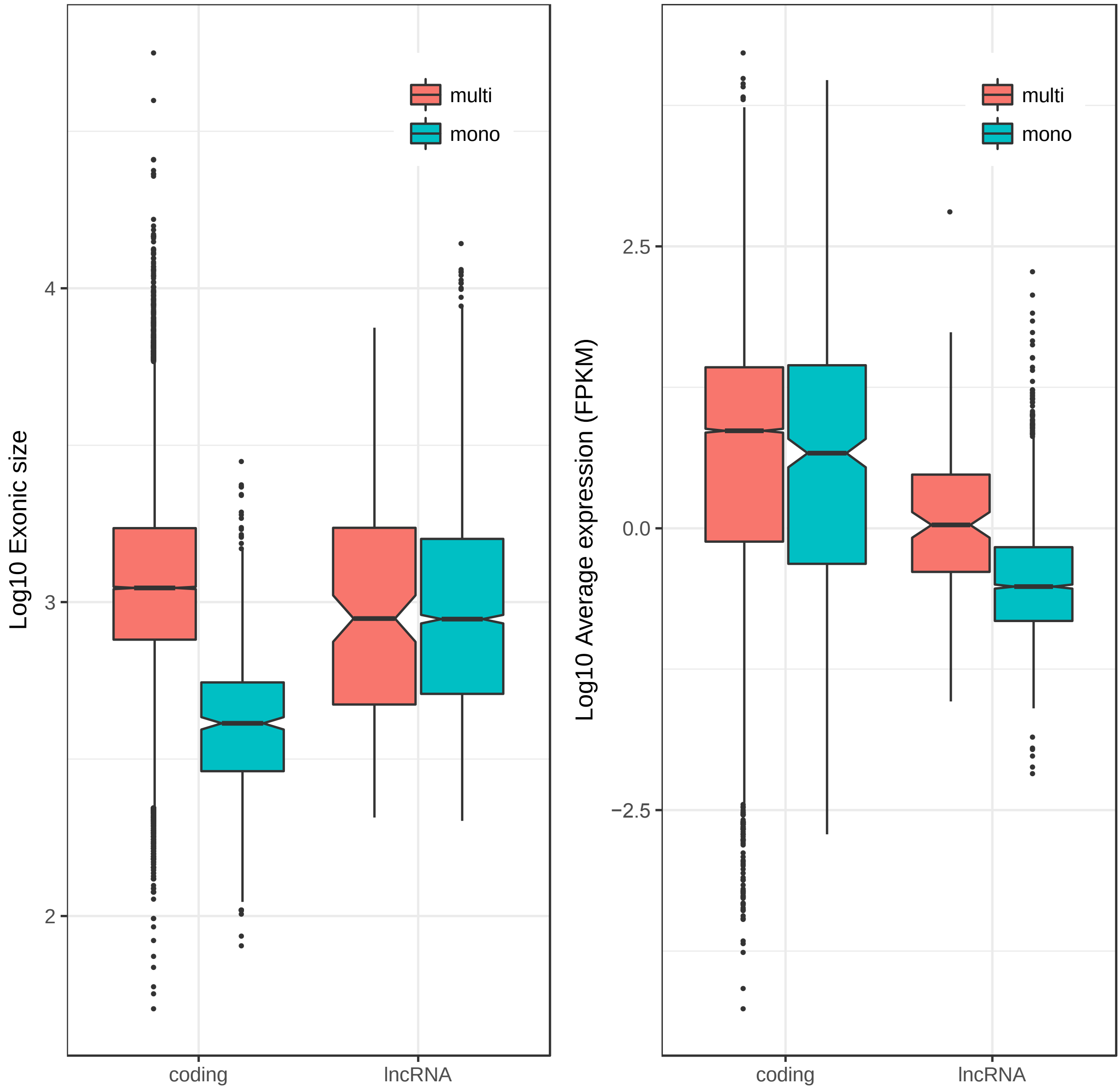

Supplement: Supplementary file 4 — Comparison of protein-coding and lncRNA transcript size (PDF 66 kb) [file 12915_2019_635_MOESM4_ESM.pdf]
