# Supplementary figures and images for: Identification of functional long non-coding RNAs in C. elegans
Source: BMC Biol. 2019 Feb 18;17:14. doi: 10.1186/s12915-019-0635-7 (PMC6378714; doi:10.1186/s12915-019-0635-7)

a)

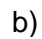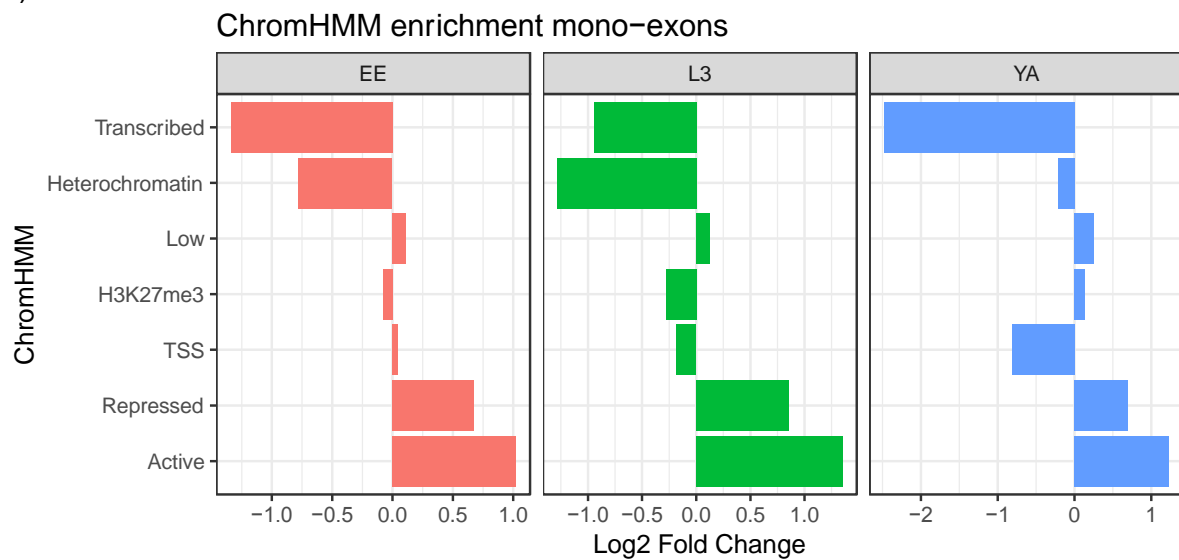

c)

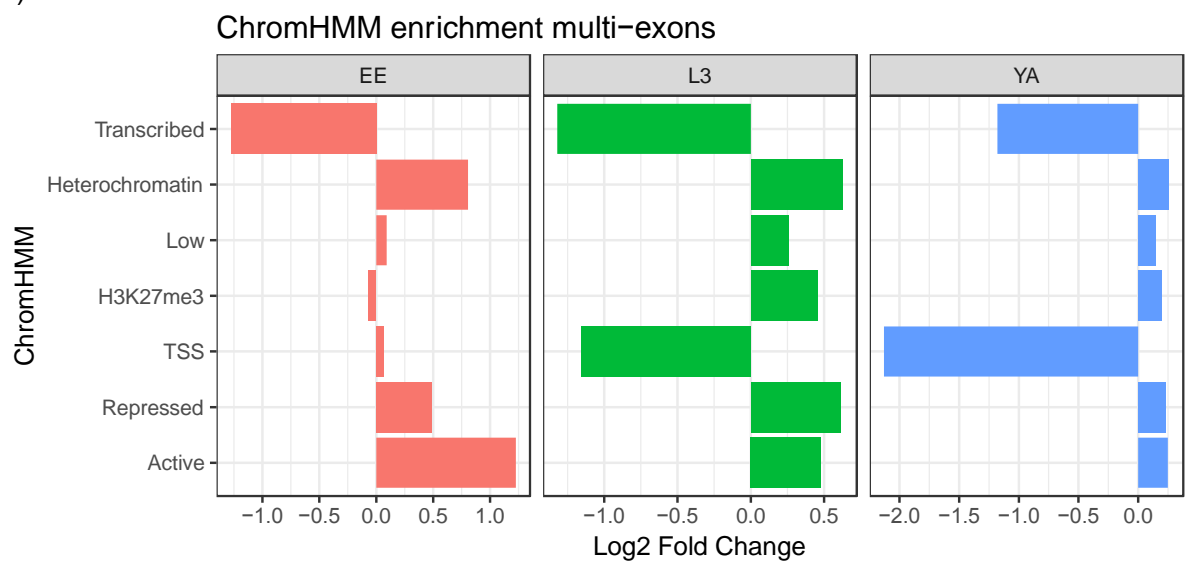

Supplement: Supplementary file 5 — Enrichment of lncRNAs (PDF 78 kb) [file 12915_2019_635_MOESM5_ESM.pdf]

**Additional file 7:RT-PCR of lncRNAs linc-239 and linc-339.**

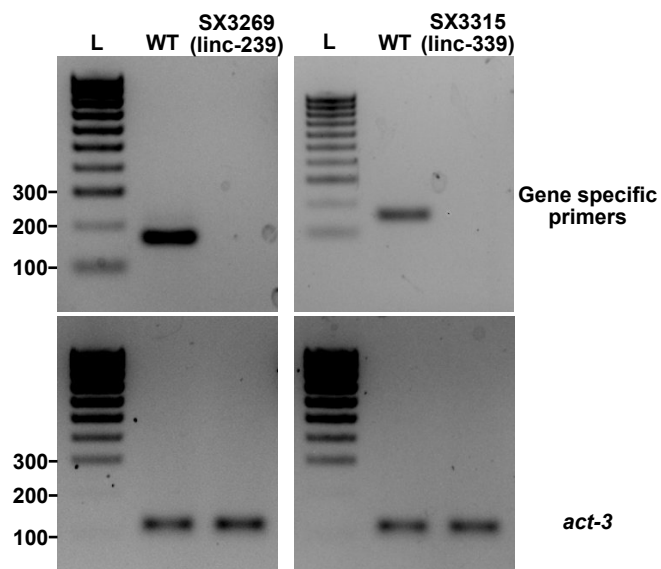

Supplement: Supplementary file 7 — RT-PCR of 2 lncRNAs, linc-239 and linc-339. End-point RT-PCR analysis of linc-239 and linc-339 in wild type and respective mutant animals (PDF 92 kb) [file 12915_2019_635_MOESM7_ESM.pdf]

Additional file 9: Genotyping of 10 lncRNA mutants

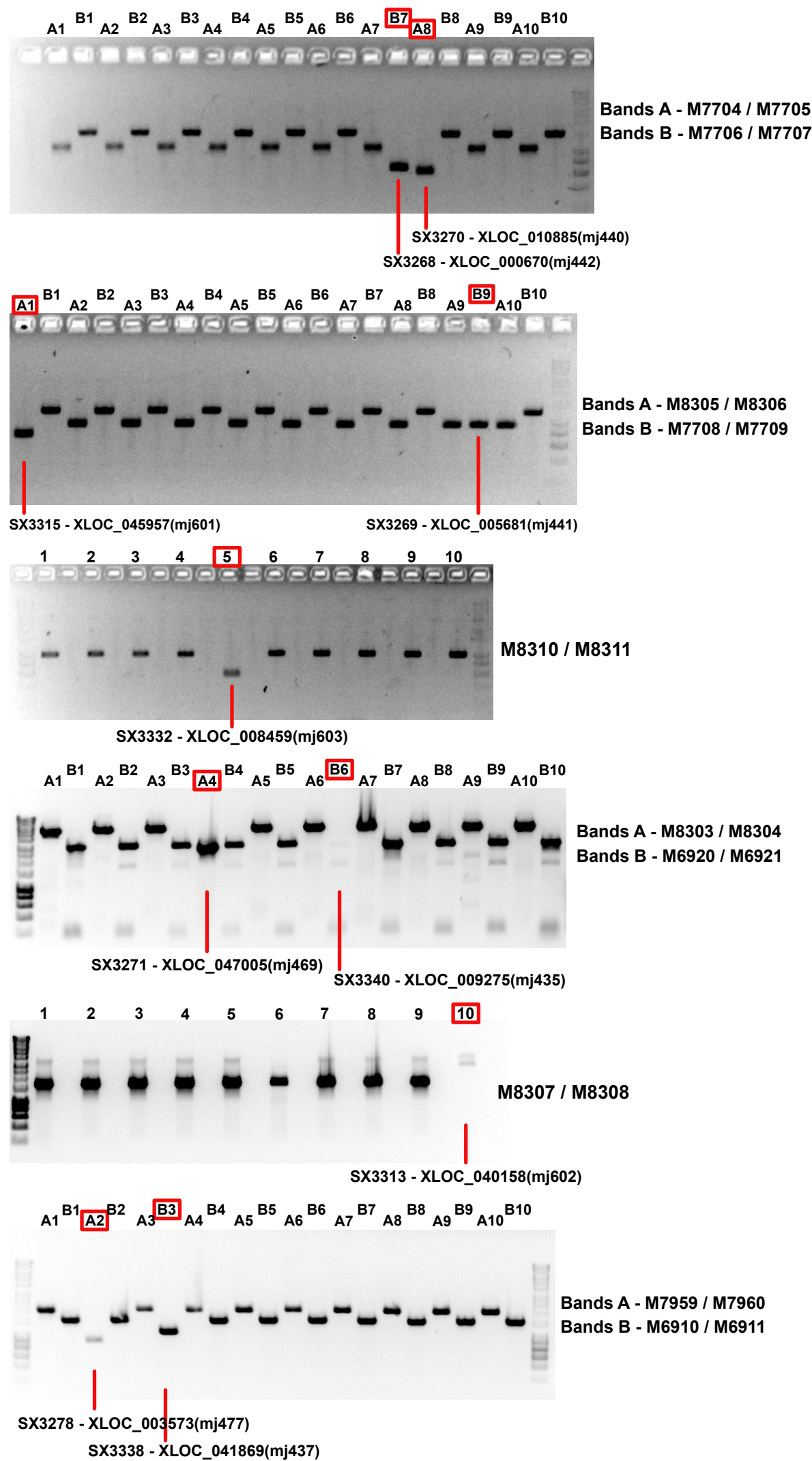

Supplement: Supplementary file 9 — Genotyping of 10 lncRNA mutants. PCR and gel electrophoresis analysis of deletion mutants for the described lincRNAs (PDF 341 kb) [file 12915_2019_635_MOESM9_ESM.pdf]
