# Supplementary material for: Identification of functional long non-coding RNAs in C. elegans
Source: BMC Biol. 2019 Feb 18;17:14. doi: 10.1186/s12915-019-0635-7 (PMC6378714; doi:10.1186/s12915-019-0635-7)
Supplement: Supplementary file 8 — Sequence of 10 lncRNAs and deletions. Full sequence information of the lincRNAs and their deletions. The legend is within the file (RTF 46 kb) [file 12915_2019_635_MOESM8_ESM.rtf]

Additional file 8: Sequence of 10 lncRNAs and deletions. GAATAG - other non-coding RNA annotationsATGTTT - protein coding genesACGTGG - lncRNA exonTTATTT - lncRNA deletion(agct) - lncRNA insertion>XLOC_003573(mj447)GACATTAAGAATAGACGCAGACAACGAGATACTCTTTTTCTATGCGTCTCAATCCCACTTTACTTCTGGCGATTTCTGTCTCTCAAAAAAAAAACTCCTGAAAAAGTAATGCAAGAGAAACGAGAAAAAATTGAAAAGAATAGTTGTACTAGGAACTAAAAGATAATCTACGTATTCGTAACTTCCTATTGAAAAGTGCAATAACTTGCTTCATCTTTTCCTTCTCTTTGCTCTCAAGAAGACTCAAAAAGCAGAGCTTATGTCTCTGATCGCCTGTCGTCTCTTCCATGTGTCTTCCAATTCCTCCCGTGTTTCTTCTCTTCTTTTTTAATACGCTCTTTGTAGTAGTGGCCCTCTATATGTGACACATTCTCAAGCACACTCACACACACAATATTTGTTTCTTTTTCTCCTACCACGTATGTATCCCTTAAGATTCTCGGATCATCCGGTTTCTCAGATTTCAGATGTTCCCCTGCTTTTCCCCCTCTAATTTTTATCTCAAATTTCTATTTTTGTCATTACAGAGTTGTTATGGATAGTACGGAAATTGATCTTATCATATGGTTTCTGGGATTTTTTGTAGTTTCAACTGTGAGTTTTTTGGAAAATTTTTAGATTTGGATTACCAGGTGCCTGATTTCTATTTATTCTGAAAATCGAAAAAATAAAAATCTGAAAAAGCATGTTTTCAAAATTCTTTTTTTGATATTCCGAATTTTTTAAGAGTACGAAAATTTGGAATATAATTTTTTCAATTCTTTGTTCTAATTGAATTATTTTAAACATCTAGAAATGTTTGGTTTTGATTTTGTTTAAAAGTTTTTGGAAACATTTCAAATTGTCTGATTATTTTGGTAGAAATTATAGTATATTTTCCCACTTTTTCGATTTCAAAAACGAAAAAAATCTATAATTTTCGCAAAATTTCTTACAAATAAAATTCCCAAATTTTAAAAATCGAAAATTGGTTTTAATTTTAAAAATTTGTTTTCCAAAAAAAAAATTCCCAAAGAAGCAAAAAATACAAACATTTTCAACCAGAAAAATTACTTAAAATATTTGCCTATATGCCTGCCTGCCTGCCGCCTATTTTTTTTTGAATTTTGTCGTAAACTTGCCCCAGGTGTTTGGTTTATATTTCGAAGAGCAAATTTACTAAATTAGACTAATTTTGAGATGGGAATTTCGGAAAAAAAACTCTGGCCAGGCAAGCATGAAGCAGTAGAAGTGAGAACACTTGGAAAAAGTGTATTTTTAAGTAATTTTTCTGTAAAAAGAAAAATTGAAGACAAATGTGGAGTTAAGGAAATTTGGAAAAATTGTAAATTTCCATCAAAACCTGTAAAGCAATTTTCTAATTTTTTTAGAAGAATCACTATACAAATTCCAAACAAATTTTTAGATAATCACAATTTTCTGTATCTGCATTCGAATTCTCCGTTCACAAGCTCACATCTCCCAAACAGACTGTTGTCATTGTGTATGTGCATGGCCACGTGGATTTGTGAAAAGACCTCCAAAACGGATAGTTCTTCTAGAACCAACTACACGAATTCAACAGTCTGGTTTCACAATTTACTGCGTTGACAGACCAATGCCTGTTACTAATCTGCCTTATCACTATGATAAGGTAAAAGTTGAAACGTGTCTGAACACCTTATTCGGCACAAAATCAATATTCCTACCGGAAACTACCGTAGCTGTTTCAATACCGGCACGTGGATTCTCGAGTATGTCGGAATTCCACATGTTCTCGAAAACTGATTCTCAACGGGAAAGTCTTTCTGGTTACTCTTATGAAATGTTATCGAGTAGTGTTCAAGCATCAAGTACTGATACTTCAAACTGTTTATTTGGATGTACATCACATTCACTGGCAGGTGTTACTGTTTATTTGGGAATTGGTGGAAGTTCAGAAAGGATTAATTCGGATGCGATTTTGATTCGAATGATTCATAATGTTCATCGATATGGGGGAAGATGGATTCCATTTTCCAGAAGTTACATTTGGATGAGAAGCTTTTTGAGAAACTGGTGGAAGCCTACAGTACCAAATGTAGCGGTAAGTAACACTTTAAACACGGTAGGTAGATACGTAGGTATGAATGCGCTTGTCTGCCTGACTACCTGAAGCATGAAAATATGAGAAAAGTTGGAGTGTTTGATGATGTCATTTGCTCATTATGGACGATATAAGTTAAGATTAAAGCCCCACAATGTATATAAGTAGATAAAAATTGCAAGCAAAAAATTTATAACTTTTTTCGGTTTTTCTTTTAAAATTCCAGATTATTTTTCATAACTTCATCATGCTTTAAGGTTTGTTAGTTCGTAGAAAAAATAATTCTGAAGTGATAATGCCTGCCTACCTTCAAAACGACCCGCCTTCTCTCATGCCTTCAAACAGGGACAATGTTCCTTGATTAAACCAATCATAATTGAAGAGGGCATTTTTAACTAAAAGGCTATGGAATTTTTAGGAAAGAAACCAAAATAAAATACGATATCAATCAATTTTCATCTTTTTCTAGGCGTACAACCGTTCCTTCTCTCTCCATCGTAATAGCAATTCGGATTCATGTGGTTTTCGTCTCCGGCAGCCATTTGTTCATCAACCGGTATTTAAGCTTCAATTTTCAAAAGGACAACATTATTTTCAGTGTCCACTCGGTGAAAATCAGAGAATGTTTGCGCCGAGACCCTATCCACATCA>XLOC_009275(mj435)GCCAATTTTTACACATTTTGTTCGTAATTTTGACGGTTTCACCGGGGGTTTTTGGAAATTCGGCACTTTCGTATTTTTAACGGTTTCTGTTTCAATTTATTGATAGCATTTCAAAAAATTTATGCTTGGGAATTTGAGGATCCTGCAGGAGCAAGAAAACGTACAATATTCGTTTGGTGGCACTGGAAAACAAAAATGGAAAACCTGTCGAAACTTGGAAAACATACTTAATTGAATTGAAGCATACAACATTACAGTTTCGGATATTTAGAAATATGCACATAGATTTGGCAATTAAAACTGGACACTCAATTTACAATTAGTCCTTATTTATCATTGGTTTGGAAAATGAGATGGAGTATAAAAATAGATAATCTATGAAAATAATCTTACTAGGTTAGTTCTTTTTAACGAGTTGTAACAATATTTAAACTGCCGTTTTTAATTTTAGCAGCCGAGAAGAGAATGTTTCAAGCCATTTTAAAAGCATAAAATTTCAGAGGATTCAACAAAGTGGATACAATCTATCAGCAACTCATAAGCCTATGGTGCATATTAAATTTTTTAAACGAATTGCCAACTGTATACAAGAAGTTACCTCCGATTGCCGAAATAATGAAAATTTTGAAAACATTTTAATTCCACATAAAATTTTACAGAAAAGGATTTTTGGTAGGTTTGCAAATTTTGGAAAACTTTGTCAGGAGACGATTTTTTGAATTTGGAAAACATTTTAAATTACCAATAATTTCTAAAAGTGCCGTATGTCAGACCTGACAAAAATATATCATAGATTTCAAAATTATGCAAATTTATGTTAGACCTGAAAAAAAAAGTTAATTTCTACACTGGAAGTGTCTTAAATATCAGTGAGCACAGTTTTTAACTCAGAACATGTCGGGACACTAAAGTTTATTGAATTTTAGATATTGTCAGGATTGAAAATTTCCAAAATTATTCCGTTAATTGAATTGTATTTTCATTTTTCGCGAATTCTACAGTTGCCGATTACAACGCAACCCTGGGTTTGGAGAGTTCATATTGCAGATGGTTTCATGACTCAAAATCTGTAGCTCCATAGAAGTAATTTTCCATATTCCAATTGACATCATATGTACACAGAAGAGCTCTCTTTTGACATCAATCTCAGTCTTCAAATTATCTTTATCTCCACACTCAACCACTCTCCGCCCATCTTGTGACTACCACCAATCTATGACAAGTCTATTCAACGATATTATGTTATTAACCACTTATTTATCTCTATTGACTGAAAATGTGCAGCAGCTTCTTTTTCCCACTCTTCTGACAAAAATACTAAGTAGCTCTTTAATACTTGAAGAGCTTAAATTTTTATATTTAATTCGGTGTGTTCAATTTTTATAGTAGTGAATCTATTCTACGAAGAGATAACTTGCTTTTAGCTGGAATGTGTGGAAATTTCCAATTTCATGATTATTTGAACTGAAAAATTGGCAAAAATGATCTTTCAAAAATCCTTGTTTCTATCTCTATTTATGTTGCAAATTTTGATAATTTTAAGGTACATATATGATATCCTTTGAGTAAATTATTGTTAAAATGAAATTTCCAAGTTCCAAGCCAACTGGCGATAACTAAATACATATTTTTGAACTTCATAGCCAATATAACCAATTTACACAGTTTGAGGTATACCCGGCTTTCTCGGAACAAAAAGTGCAAATGAAAACGCATTAGGATACTGCTCCACAATACTTGGTACACCTGAAGTTGAGAATTTGTTTTGTGAGTTATCACTAAAAAATTCGTACAGGCCCTGTAAGAATCCAACGGACTCAATTTTATCCAGGACCTCGTTACGAACACGCTTTCCCAATTACTTTAAAAGGGTGGTACCAGCTTTCTATTTTGAGCAATCTTCTTCGAATCCTTGAGTGCTTCTTAGTTCTGCTTTCATAATCTTCTTCTTCTCCAATTCCATAAAAATTATTGTGGTGCTCCATAACTGCAGGTACAAAATCCAGTGACGGGATATAATCCGCTGAAAATCTCTCAATTCTCATATGGTCTCTGAGCGTCTCTAATCGTCTATTCGCGGCATGTATATTATGATATTATATTTATGATTAGTATGATTATTAGTGTTATTAACACGTTATTGAAAGTGGTGGGCATCAACGACAGCAGTAGCAGCCGAAGAAGAAGATAATTCCTTGGATATAGAGCCAGAAGATACTAATATTAATCGGAAGTCTTGGCGAAGGAAAACTTGTTTTTCCGCGCTGCATCAATATTGAGCATTTCATATGCTAAAAGCTGAGCAAGCTCTGAGTATAACAATTTACTGTAACTGATCTAAATTTGATTAACCATTACGCTTAGTTTTCTGAAAAGCATTTTTGATTTCCTGTTGGAGAAATTCATCAGTTGGTAAAATTTTGCTAAACAAGCTTGTTCTAAGCCGTGTTCCCTATTAGCATAGAAATTTTACTGTTCTATTTTGGAAGTTATATAAATTTTTTTAAAGAACATAGGAAACAAACATAAATGCTGCGAAACTAATTTTCAAGTTGCTCGTAAGGCTACCAATACTAATATTAAGCTTCTACAGTAGTTAGCTATGGCTTCTCTTTGGTCAGGAATAAGAGCCCGCTGGGAGCACAATCAGGCAGTCTACTTCCCAATTTTTTTTCAAGATTCTCAAATCCACATTCGCCTATGGTTGCTGAAAAAAGAAATTTTATTATTTTGAATAAATTGTTAATTTTAAATTAATTTTCTAGAAGTGCATTTGCTAATCTCAAATTTCAAGTAAATTTGGAAAACTAGCTAATTAGAATAACTCTTTCATAATAAATCCCTAATTTCTGAAATATAATCCAAAAACTAATTATCCTCTTAATGTATACAATTTCTATCCCTCTTTCTCCATCTTAAACATTTGATAGCTCCTTCAAGGTGTGCCCCCCCCCCCCCCCCCCCCCCGTCCGTCTAATCCCTTCTTCTTCGTCTCTGTCCTCCTAATTACACTTAAACCAAATTCCACGTAAGTAGCCATTGGTGCCGCCCGTCTCTTGTTTTGCTTGTCTTCATCTACTACCTCCACCTGGCTTTACCTCTCACACATCTTACTCATTTATGTGTGTATTTGTCCAAGGGGCCCCACAGACCACCACCACCACCATCATCATCATTTCGGCTCTTTTCCTTTTATGAGTGACCCATAATATAAAATGTCTTGTGTTACTTAGACATATTGGGATTAGTAAGTTTGCTGCTAAAAGTGAGTCACAGCAAATAATTTTCCGTAAGGGGCCATAAGGGAACGTATCTAAAGTGGCCTATCACTTTTGGGATTATTGGCTCAATTTTTTCTAAGTCTTTAGGACAACTACATTTTCTTAAAATGATCCAGTGTGTTGAATAATTTTTCTACCGCTTATCTATACTCGTCTATAATGTAACCATACAATATTCCAACTTATGTTAATCATAATGTTAGGAATGTGCAGAATTTGGTCCAAGAAATATCAACTCATCAGAAGTTGGCTAAACTTGGGCAAAAGCTGTGCAAAAGTTTGGCAAAAGACTTTGGCAAAACTTGGTTTTAAGATGAGCTAAAGCTTAACCCAATTTTGACCCAACTCTTGCCACACTCTTATCACAAAATCAAGAGCCTCATTCAATCGCAAATTTGTCAATTTTTTATATAAATTTTTGAAGAAATTTTCCTCCAAAAACTATCTACATATTTTATTTCGAAAATCCTAATACCATTGTTTTGAACTTTCAAAAGCTCCCGTGACTCATTCATTACAAGTAAAAAATAGCGAGTTCTCTAAGAGAATGCCTTTCTGCCTTTGCTCACCCGTTTACGCTGAAGAAGAGCCATTCAACCATAGGAGCTTGTCCTCATTATCAAAGCTAATTAGGAGGCTAGTTTCCTTTTCTCCCCACGCCTATTCCATTATACCAGCTGAATAGTCTTATTTTATTTCCACTTGGTTGCGTTCTTATTTCAGATGCTCACTAATTAATTATCGATCGATAATTGACTATAGGTTTAAGATAATTGTAAAAACTACACATTCAGTTTTACAGTAAAACTCTAATTTCTGAATAAATTAATTGTTGAGGCGTACTAATTTTAATTTTTTAAACACCAATTAATTCAGTTTCACTTAACCCAATTATACGTCTGCTATATTTTCCCATCCAAACATGCAATTTGCAGGCACAATTTATGTCTCTCGTCCCACTCTCTCACAGAAAATTGCATTTTCCATCGATTCATGTGTATGTGTGTGTACGCAGGGCGCGTTAGCCCTAATTATCATAAATTGCCACCCTTCTCTTCGTGCTAAACCGCACCCAATTATCGGGCAGATGGCCTCGTCGCCTCGCTTTTCTCATTCTTTCTTTTTACGTAGTTCCGTGTATTTTGACTGTTTTAAAGTATTCTATTGCTTTGTTACAGTGTGGACTCTAATTAATTCGAAAAATTAGTTTTATAAGGGAAAATTACAACATTTTTTTGAAATTTATTGTTTTACTTAAAAATTTAATTCAACAAAATGTTTGAATACCTTGAACACTAAACACCAATAAAAATATTTTTATATCTTAATTACTTATAAAAATCGCTATAAAAACTCAAAAATAGACGCCGCCAAAAGATTGTTGACCACTAGTGCTATCCTGTACCAGTATAATATTTTGGTAAATATTAAATTTCATCAAAAATAAATTTGAGGAAAATGACAATCACTGATAATGAATGATAATTGAGCGTTGAATAAATATATCAGCAAGTTTTGATTATCACCTGTAAACACACACAATTTTGTCTGGGCGTGCGTTGGTAATTAAACATACTTTGATTATTGCGAAACTAATTATAAATTATATTTTTTCACACTTTTTGGTAATAATTACAAAGTTCAAATATTTTTTAAGAAAAAAAACAGTATTTAAACTTTTAAATAAAGTCTAAAAATATAGCTGCAATAAAAGTGGAGAATCTCTGAAAATTCCTTATATGTAATTGTCTGAGAAGGTTTTGCGGAGCCAATGTTCTCTGCACAGACTAGTTTTTCATGGCGGCATAGATCTACCAAATTCTAATGTACATAATTAATTTTAGCAAACAACTTTTTAATATTTAGTCTCTAAAAATGATCCATTATTCCTCTAAAAAACATTTTTCCAGTTTCCAACAAAAGCAGTCAAACCACCAAAAAACGTCTCAAATTTCAATAGAAACGGGCAGACACCGGGGAAGCATTTCTCGAAATTCTATTATTTTGTTCTTTCCCTCCTTCTCTGTCTTATTCGTTCGTTTGTTTCCAAAACAAAATATGTATTTATACAATATTCTTGTCTACGTCTGTTAACTCTCTCACACCGGGGTTTTCGTCTTTTCAAAAGACATAATTATCGACCAATTTGGAGGGGAAGGGGAATGGAGCGCGTTTCTGCCAATTTCGGTCACACAATTTAGCATAAAGGACATCGATTTTTTAGTTAGCACACATATATTCTAGGAAGATACGATATTACATTTAGGGTTTCGAAAGAAATTACCACTTTTCTATATTCTAAAGATACTAATTAGCAACATTGAAAAGCATTAAAAATAGTGTTTTTTTTCAATTAGTCTTACTCTATGCTCTATTTTGACAGCTCATATCTAAGTTAACTTCGATTTTACAAAAGTATGTTCAACTATTGATATAGGAAAATTTTTGGCACGTATTTTGTTTCTGCAAATTTTTTTTTGATAAAACCAAATGACCGTGAAATTAAGGCAATAGGGGTCGTAAACCTTATAGAAAAAAATTCGGTATATTTTTTAATGATTTTTCCCCAATTAAACCCGTTTTTTTAAACTTGTTCTATTTATCTAGTTAATCTTTTCTATCAGAATGCGCTATTCAAATTATATCAGGCATTTCTTCAGGCATACCTCTTTCCTGCCTGAATTTTTTCTTGAATTCACATGATTGATAGTATAGATTTATGCACTTTTCATGTTCACATTTTAATTAATTTTTTTGAAAGTTCAGCAAAAAATCTTGAGCTGTACTTTGACAGTGAGCAATGCCTCGGCCGTCTCAAAGCAATTTTTTTTATTTCCTAATACTATAATTGCATTGGCTAATTTTTGTTACCATGAATAAACTTTATTTGTAGTCTTATCAGAATCCTGATAATCACATCTAGATTTGATGATTAGTTCTATAATTTTTTCAGCTTGTTTGCTCAGATGCACAACTGGAAAACAGAATGCTGTTGATAATTGCTCAGTGAAGCAACCTTTCGTTATGAGCTAATCATATGATTTCTTGAAAACTCATGCCAAATGAGCAATTTCAGAGAGTTTTAAAACAGTTCTCGCAGTGATTTTTCCATTAGAAACTTATCAAAGAGGTTTGCAAAACATTAAAAAATTCTTTGCAAAAGAAACCATTTACATTTATAATTAATTATGGGAGCCGAGCAAAACAACATTTTTTGCAATTTTTTAAATCCAAAACTTGGAATCTGTATTCCACGAATGTTTCTATATTTGCAAAGTGTGTGAGAATATCAGAAGCTGAACAAAAATTCATGTAAAATCTAGATTCTAAATTATTCTGGTGTATTAAATTGAAAGTATAGCAATTTTTACCAGTTTTTGCTATGTTAGCTATCATATTTGAATGTTGCAACTCACTCCTCCAAAACCCTCTAAATCAAATTCCAAATCCTTATGGACCATTCAATGCCCGTTATTATTCACATTGAATCTTCTAAAACTTTCCCACAAGCACGACCAGCACCACCCCATTCAAAGCTGCTCCTTCATCAGCATCCATCCGAAAGCGTCCATCAATGTCTTCAGGCATTCCATCTAAATTCATTCACATTCAAAAGCAGTAGGATAGATGTGCGAGAGAGACGAGAAATCCTCCGATTTTCTTCAAATAAACATAGAGAGTGAACGACGACTAAAGGACCCTTTTCTCCTTTCTCTCGTCGTCTGATCCTCTCGTATCTCTCGTCTACCACCACTTAACCACCAACCAACCACATCTTTCTAAACACACAGCGGCAATTAACTAATCGCTGAGAACTTTTGGTAGGGTTTCGTAGTATGTTGTAGAGAAACTGTGAAGGCACTTTTTTTTGGAAAAACTTTAGTGATATTTGATTTTTTTTTGCCCAAATTTCAGATTTTTTTAAAAATAAATTACTGCAAGACTATAGTCTAATCAATGTCTAGCTAATTGTTCAAGAAGACAAGTTCTACAAGTTATCCATTTTTGCCGTATCATCTATTATTATCCTGGTAAAACCTAAGTTTGAATCTTTTGTAGTGATCGTGCCGCAAGTTATTCTAGGCCTGTGATTACCTATCGAGTTATGCATTTTCATTAAAAAATACCATTCATTGAACACTTAAAGTGCTAAAATGTCTAAATATCGTAGTAAAACCTTTTCAAAACTCCAAAATTAATTTTGGAAGTCGGCCAACTTCTAAAATTAATTTTGGAAGTCGGTCAACTTCTAAAATTAATTTTGGAAGTTGGTCATAATTATTTTCCAACATTAATTTTATTGTAATTGCATTTCAACTGTTTGTATTCAAAAATTCAAGGGGTTCACATTTTTTGGAAAATTTGGTATTTAGACGATATTCCACCCTTAACTTAGTATACTTATCATGGAAATCAAATAAAGGTTTCAGTGCTAGAAAAAATCTATAGCAACATTTTCTAGAAATTCATTTTTGATCTACCCGGTAAACTGAACTTGATCTCCTTTTTCTTTAGAAAATAGCTCTCCAATTATAAACTTTCCTTTTTTGATTTTTCACGAAATCAAGAGACTTTTCACTCCAAATCACATATACTTTTCTCCACCTTCTCCCCCATTTCAATTATCCTCCTCATAATCTTCTCCACAGTCTTCCACTTTTTCTTCCGTTTTTCTTTCCTCTTTCTTGCCTCTTCCTCTCACTGTCTATGACTCCAAAATGTTCAATTGAAAAGAACGAACCTTCTCCAAGCGGGCAAATTGGTGAGAGTGAGGCGTTTGGTTTTATTGGACCTTTTGGTGTATGCCAGAGAGCATTCTTTGTGATTTGAGATTAAAATGTTCTCCAATTGTTGGCACTTCTACTCTCCGTCTCTTTTATAATTCTACGGAGAGGCAAGAGATGAGATAATTAAATCAATTATTCCAACTAATTAAGTGCCTCGGTCTCTCCCCATGTCCTTCCATCCACGTATTGATTGGTTGAACAGAAATTTCTTAGCTTTTCTTTCTTTCAGCCTAATTTTTCTTATTTGATTGAAACGTCCTCTTCTGTAATTTCGAAACGTTCTAATTTTTAAATTGATTTTGTATTGTTTTTAAAAACATATTTTAATTAATTTTTTCAGGATACTACCTCAAAAATGTCCG>XLOC_000670(mj442)CTTTTTCAATTTATTTTGCACTGGTAATTCGGTGCTCGACGAATTTTCTTCTTCAGACATTTTAGCGTATAGTATTCGGATAGAAATGTGTAAAAATGATGAAAAGCGTAGCATTCAATGAGACCCATATGGAAATTATGAAACCAGAATAATTTGGTAATTGTATTAGTTTAGAAATATTTGTTTAAGTTATTGTGTTCAATTTGTTTTGTTTAGGCAGCAACTGATTTGGATGAGAAGAGTTAAAAATAGGTTATTTAATCAATAAAAGCAATATGAAAAGTATTCCTCAGTAGGGTCTAACAATAAATACACTAATGGTCAAAATAATATAACTTATTCCAGTCTAAAAATATTCACGAATTTTCTTCTCCTAAATACTAGAAATTCTTAAATTTATCAAAAACCAGAAAAAATTGCAACATCTACAGTCAGTTTAAATTGTTTTTGAATTTTGGTATTTTGGTATTTGAGAAACGTTCAAATCAGATTTTAAAAATATTGGATAGGTATTTTCGCACTCATACATCTTTGAAATAAAACATTAAGTTTTTTCTCAATACGTTTGAAAAAAGTACAGTACTACATAGTATATGCACACATTTTCCAAATATATATTATCAACAGAAAACTATTCTCATCATTCTATTTTCCCAATATTCCCATCCACTTTGAATCACTAACAACAATCATGTGATTTCCTAAATGTTTTCCTTTTTTCAAATTGAAGAAAATCAACGCTGCGGAACACATTTTCCCGAAAAAAGCCGTCCGAGTTGTTCCCGTCCACACCTTTTTTCCCTCTCAAGAGTTATTCTTTTGTAGATTCTTTGGATCATTCACTTGATAGCCTAATAGTGGGCGTGTGTTTGTCTGTGCTCCGTTACGTGAAAAACGGAACAAATATTTTTTGAAAAAAAAACATTCGAATTTTTCCGTCGCGTCTCGAAATGCCCATTTCTCCTGCTGGTAGGTTTTTATAGACCTCTATTTGAAAATTTAGTTATGAATGTTTAAATAAGTGTCTCTAAAACTATAAAAAACCAATTTTTAGAGGAATCTTGGTAGATGCTCACTTTTCAGCTCACATTGATTAAATCCTCAAAAGTTCACATATAATCATTGAAATTGTTTTTTGAAATAATTTTTTAAAAAGTTATGTTTGACTCATTCTGTTTTGGTTACTTTGGCAACTACCTGAACACAGTCTCCTGTATCAATTGCTCTTAAACTTTTTAATCGAAATTTTCAGATTCAACAAGCATTGCTCGTCAAAAACAACGTCGCATCGAACCACTTGTAATGCTTGATTCTCGGGATTACGGTAGTTGGAGGAATGGAACAACTCCATCGCCGAGCACAAATAACAAAAGCCCTCCGGTATTTCAAAATGAGAAGAAGAGAGTTCATTGGCGGAAATAATTCTTGTTTCAATTTTTTTTTGTATTCTCTGTAAACTGTGTACATATTTTGTAAATTGGATTACTAATTTAAAAATAAATACTTGTAAAATAAAATGAAATAAATAAATAACTGTAAAAGCGTACTTCAGGTAGATCCAAAATATTTGGAGTGGATTTAATTTTTGGGTTCAGGTCAAGTCTGTGCGGCAAACGGCACAAGGTTCAAGGGACAAGCAACCGAAATCATTGATACTAACGAAAAGTTGAAATGCAGTAAGAAGATCAAGGAATTACCGGACAAAATTATTGCCAAGGAACAAAGGCATACGTCTCAGCTTCATTTGCATGATCCTGTTCAAGTATTCCGTGACATCATCAACCACCATCACGTGTGTACCTCATACCAGGGCTGTGCGGCATCCTGAATTTTCGGCGAACGTTCGCCGACACATAAATTTCTCATAATTTTTCGGCGATCGGCATTTATCGGCACTTTTCGGCATCCTTAAAAAAAAAATTTTGTGTTTTTTTTTCAATTTTCTAAATCTAAATAGTTGAATTCCAAAGTTTTATAAAGAGTGTCTGGTTTGAATTTTAAAGCATAGATCAATTTCATATTTAAACGACCAGACTGTTGGGATAGTCAAAAAAGGATCCCAGAGGCAAGAATGATTCAAAATAATTGTGTCGACTAAAAGAATATGGCTAATAATTCAAATATGTTAATTTTTGGGAAGTGTTAGAAATACAGGTGATTTGGGAGTGTTATGATACAAGAGACACAGAGATGTCTTTGGTTGTGTTTTATGACTATAATGTTTGTAGATTTATGTTTCAACGGATGAATGTGAAAAACTTGAATGTATATTCGATTAAATTTTTAAAAAATAAATTTTTCACAAATGCCGAAAAATACCGAAAAAAGTCGAACAAAAGTCGACCGTTTGCCGCACATCTCTCATACATATGAACGTCATATTTGAATATTCTCGTTTAAGCTGATGTGCACGGCATTTGGAATATTACGGGAATTGCCAAAACCATAGAAAATATTTTCCTAACAGCAATCGAAACTATTTGATTCCATAACTCCAATTCCCTTCTTTAAACTAGAAATTTATAAGCACATGCCTGTTCTAAGTATCCATCAATCAAATGCCAAGAAACCGCAAAACAAGGCGAAAATTATCAGAATTCTCAACAAGTCATCCGGGCGGCTCCCAAGAGGCAGTGCCGCGGCGGCAACACGCTCATCTTAAATGAAAACCCAAGAAAGAGACGCTATATAATGTTTTCCGAAATATCAATCATGCTTCTCCTCACACATCAGCTTCTCGTCTTCTCGTCCTACCGTAGTCGCTTATGAACCGAGCCGCCTCCCTAGAGACTATGGGATCCTCAGCGGTAAGTGATGGGCTCCGCCTTGTAAAATGCAACATTTTCCCTCATTCAATATGAGCCGGATGATTTTTCGCATATTTATCATTTTTGTTTCAAAGACTCCTACAGTACTCTTCTAATCGTAAACCTTTTCAATTGACAATAATTTCAGAATGATGTTTTCCTCAATAGATTCTCTTCTTAAAATCTCAACTTCTTCTCAAAATGAAGATCAAAAATTAGAGAGTCACCCCTCCCCTCCATCACAAATTCCAAATTATTCAACCAGCTGCTCTGAGGAATTAATGAAAATGGCGGCAAAAGCAGCCCAATTTGCTGCTCAAGCCTCGCTGGAGAACTCATTTTCCAGTTCTA>XLOC_047005(mj469)ATTACAAAAAATATTAATAAAAAAACAAAAATGAACATGCAAAATAATATACTGGTTGGAGCAAGAAGGTGTGATGTGGAAACGTGAGGTTTTATCTTCGTGGGGGCTTTCACATACCCGCGACCAACTAAAATTACAACAAATGTCATCCCAATGAGACACAAATAAATCAAAAGACACGAAATACCGAGAGCAATGCCGTCTCGTGAGGTAGAATCACTTTTGAGTCCGTTCCCTGATAAAAGTAGTGAGATCATGATATTTCATGGATGTTTATTCAAAAGATATATAATTTGTTGCAATTTATGTGGATAATTGTTCATTTGATAATCAGCGTGAAAACTCACAGTGGGAACTAGATGAAATGGAAAACGAGAAAACATGAAGTAAAAATGAAATGAATGATAATTTGGACGAGAATTTTCACTGGGTTATATATATTTATACAATAATAATTAAGCCTGATTAAATATCACAATTAAACACTCCAAAGTTTTTTCAAAAGTTTTAAAAATAAGTGAAGGTTCACATTTTTGTTCTTCAAGTCTCTATTCACATTGGTCAGGCTTGATTCAGAAAAAGTTCCCACGAACTACTACGTCATCGGAAACTTAGTTATTCCAACAATGAAAATGTCAAAATATGTTTTATGAAGAGTTTAACCTTTGCATATATAATTAATTGTCAAAATACGATTCCTAAACTCCGAAAAATTTCAGTTTTCCATCAATTTACTCACCGACGCGTTCCAAATTCCAATTTCAATATACTAGCCCAGAGGCAATATAGATTATAACTTTGCCCCCCCCCCCCCTTTTTTATTTTTCATATCGCCCATATTTGTAAACCAACCTCTAAATTGATGCCTGAGCTTGTGTGAGCCATTCCTCGTTTTCGTACACTTCGGTGTCTGGAAAATTGAAAAGTGTGATTAAAATGATTAGCTTTTTTTGCACACCTAGAATTAAATTGGAAAACTATGTGTCAAAAATTTACATGTTTTTCGGAAAATTCCATTTAATTGCAGGGTTTGTGTAATTGGTGTTGGTTGGTGTCTATAGAAGAGTTGATAAAAACAATTAATGTAGTTTTTTAAACTATTTCGTAAAGTTGCAAGTTTTTTGCTATAGTTAAGTAGCATTAATTCAATATCGACTTTAAGTGGGCTATCACTAGTGAGGGTTTTGTCGAAAAAATAAAATACTGAATAACTAACTAACCTTAGAATGTTCTAGATGTTTAAACTTTAAGAAACTTTCACAAGTTATTACTGATAGTGAAATAAAAGACAAATCCTAAAAATCTTTAACTACTTCTCGAACTCAGGTATAACTGAAGTGAGCTCAAAGTCTTCGTACCCAAAATGAGTTGTCTAGCTTTTCTCATAGCTAATTGGGAAAGCATCTAACGTAAATAAGCCGGAGGAAACGAGCTTTACATTGAAGTTTATGTCCAAAAACCACTTTGGGTTAACTTTAAGTCCTATTGCAGCTTTTCTGCATTAAAAAACCAAAGAAATAATTGAAAGAACACTGAAATTGGTACCTTCGGAAAGACTTGCGTACAGTCCGAAAAAATGAAGTAGGTGACGAGGAAGCGAGCATGATGGTTAGTGAGTTTTAAATAGTAGTGGTGCCAGAACAAAAATAGAAAGAATAGTGTGAGGTTGTTCGTATTGCAGACAATGTCTCAACAATTGCATTCAATTGATGAGCCGCTTCATAGCAAGATTCTCAGAGAATGAGACAGAGGAAGTGCCGATTTGGTAATTGTTTGTTCGTCCCAAAACCCGACTTGCGTTGGCGTTAGCGACGAAAGATTCTCTTCGTCTTTCACTTGTCCAGATGAATAATTGGTATTGCCTTTGGCGTTCTTTTTTTTGTGACTCTTTATGTGCCTTTTCCAACTTCTAGATGCAAATGTTCCATGTAAAACAAACGGCTAGGATAGGATAGTGCGGTAGACTTCAGAAGCACAGGAAATAGTAAAAAGAGTCGAGTGGTGGTCGGAGGTCGTATACCACTTTCGATACTGGTGTCGGTGCAGATATGGAAATGAGTGGGCTGGCAAAAAGTTGAAGGACAGCTTCAGAAAGGGGTGCCTTTCCCTTTGCGTCCCACTTGATCAAACGTCGAGCCTCGGTTTCGTTAGCACAACTTGAGTGCCCAAATTAGTATGCGGTGGGGCTGAGGGCGCGCACTGAGATTATGTCGTTGATTGAATAATACTCTGTTATGCTTCAAGCACTTCACCAGCTTGGGATCCGTTCACTAAAAGAAATTGAGAATTAGACCAACACCTTGACAGCGTTACTAAAATCAACACCAGACAATTATGAATTCAATAGTAATAGTTAAGTTTGGGGGATTATGATTGATTTTTGTTGTGAAATGTAGTATGTTTGAAAAAAATTATGTAGTCCGGCTCAAGTAAGTTTGTCCGAGATGCGAATTTTGTTATTAATTGGAATTTTTAAGTGTCAAAAATACGAATAATATATATATATAGTTCACAAGTTTATTGTTCAGCTACATTTTTAAAATTTTTAAATACTGAATATTTCCAGTTCAAGACTTGAGCTTAAAATTCAACTCTGAAAACTTCATAAGCTCGGGAATCCAAGCCTAAAAACTTAAACAAAAACTAGTGTCTGAGGTTCCAAACCTATGGACCTGCGCTTTGTTCAATATGTGAGGTAAATTATCATTATGCTCTATTTAAAAAAAAAAAAAAACTATACTTGCTTTTTCTAAAGAAATGTTTTAAAAGCGGTTCATAGAAGGGCAAATATTTTTGAATCTCCTAAACGAAAACGGTTGTTCACTTTTGATAATAAAACATATGTTATATTTCAATTTTTAAAAAATGCTTGAAATTTTATCTGATATTGAAAAAGAAAATTGATAAGATTTTCAGCAACTTACTGAAAAATACACCGCTTTAACTGAAAAAAAAACGTCAATATGCAATCAGAAATTGTGAATTTCCAATCTCAAAAAAAAAAGAAAAGAAATGATCAATTCCTTTTAATAATTTATGTTTCAATTCTAATCAGTCAAGAAACCAAAAACGCCCTCAAACAATACTTTTAATCTCAAAAAGAAGAATGAATAAATTCTCGTAGATAATTTATGTTTCAATTCTAATAACTCGAAAACTCAATAATAATCTGGTTTTGATTTATTGGAGCCATGAAAGAAAAAAAAACGATGTATTCTCATTTCCGACTTAAAAAGCCAGTGGCTTTGATACCAAAGAAAAGGCGGGAAATGAGAAATAACAGCGTGTTTCGCCGAATGCATCTTTCCTTTCTTTGCATCGTCAGACGCCTAGTAAAGGTGTGCATATCAGAGCCAGCCACCGCTCATTAACGCATCGCAAAATAAGCGACAAACCTGATGAAACTTGGGCATTCAGGCGCAAGAAGCTTGCTTAACAATTTCGACGAAAAAGAGCAAATCGGGCAAGTGGAGACGCTGGTTTCGGGACGACGGCCAGGCCCGGGTCAGTTTCTTTGGAGCTGTACCGATTAAACATGCACATACACAATCGGCAGGATGGCTCAAGGAGGTCAAAAGAAGAAGAAGAATGTACAGATATAGTGCGCGCCGTTTCCTTTTGTTTTGAAAAATTGTGACGGAGCATAAACACGAGAGCTCAGAAAAGGGTGTGAAGTTACTCATTACACCTCCGGGAATTGCACTTGCTATCAGCGGCGGGTTCGCAATGCTTTCATCATTGAACACGTTCATTTTTCCCCTTGAAATTCCATGCATAGAAACTGAGTTGAAACACAATGGCGTACGTTTTTATACATATAAATGAGCAAAAATGAGATGAAACAAGGCGACAAAGCATGTGAAAAGATAACAAAGCCCGAATGTAGGTGTGAGAAAACTGCATGAAAATGAATCAAAGAGGAACGAATGAAGAAGGAAAGGGCAAATAATTTTAAGGGTGCTGAGAAGAAGAAAGGTAGGGAGATAATATGAAACGAGATATCCTCTAGACTTTCGTGAGAATTTAGGGAAAAGTCGAGAAGAAAATGAAGGACGCCGGAAAGATTTTTTATAGGTTTCTGGTTTATTGTTTTGTCGTTAAAACAAATTTTGAACACCAACCTGATTTTCGAAATTGATTTTCAATTGAACATTTGAACGGGATATTTAGCTTCGGTTAGAGGGCTTGGCAAGATTTCAAAACTTGACAGGTAGGTTTTTTTCTGACCTTGCTATTCCATGTTGACACTCCAATCCGAGAGATAGCCAGAACCTCGGAGTGACAAAAAAGACGACGGACGGAACGAGAGGAGGGATGAGCGGTGTGAATATAACAGAACAATTAGTACGAATAAAAACCTGTTTATTGATCAGGAAAAACGTAGAGAATGTGCTGCAGGGCAGCCAGGAGTCGAATAATAATTAATTAGAAGGAGTACGGCATCACACGATGTGAAAATCGGAGGAGAGTGACGATAATTGCATCATCAAATTTAGGCCAATAATTAATGTGAGCAATGGCTAAAGAGAGCGCGCTACGACACTCCGTTGTTGTCCGTGGAGCGCGCCTGCATTGCTTGGCGGCAAATTCAAATAATAATTGTTTATTAAATGATTATTAGAGCGAATGTTCTTGGAAGAATACGGAAGATATTGTGAGTATTCGGCGGCGGATGTTAATTGTGGGATCGTGGGTGATTCTGACCAATTAATTAACTATACATCCGCAAAGTTGGGATGCGCCGGCTCTCCGGAGCCCAAGTCACAACATTCCGGGGGGGAGCTTGGAGACTCAGCTTATAGCAACCAGTTAGTCCTTGAATAATTTACAGCGGGCATAGGCCTCGCGAGCGTTTCCGTATAGCTCTTTTTGAAGATCAACGTGTTTGTTGTGTTGTCGTGGTATTAATGTTACGCGGATGTATGTGACTGGTTGTGTCGGTAAGGAGTCGATGACCTCGAAGAAATCAACAAGAAACATCGGTCGGTACGGTGTCGTACATGCGCCTGGCCGGTAGGTGGTGTAGGTGTGTGGTCCTTTGTACGACTTGGAGTAGATATTTTCTTTGTAGATTGTAAAAAACCCACTTTACCGGGAGTGAACTCTCGACCGGAAAGCACGGGAAAAAAAAAACGAACTTGTAGAAAACCCATTAGCCGGGAGTGGAACCTCGACCGGAACGGATGGGAAAAACTGAGTATTGACTACGGAAAAAACCTGGTTAGCCGGGAGTAGTGTATCGACCGGAGTAAGCCAGGAAAAAAAAACTTGAGCGTAGTTGTGGTTTACGTAGGAAAAAACCAAGCTTGCCGGGAGTTATAGCATCGACCGGGGCAGCTGGAAAAAAACGTCTTCTTTCTGCGTTTTCACGAAAAAAATTGCCGATGTCACCGGGAGAAATGTCGTCGACCGGAGGAAACAAGGGGAAAAAACGTGAGTGTGCCTTGTAGTTGTTTAATGGATTGTAGGGAAATCCATGATTCCTATTGAAGGTATTGCAGCCGCGAGGGGCTGTATGTCCGTGGATGGATTATAGAGGAATCCATGATGAGAGGTATGTCCGTAGAATCGTGATTGGTCTGACGAGAGTGGAAATTTGTGTTTTCCATGAGCCAAGTGCGTTTTTGATGCTCATATGTTACGTGGTAACTTCGGTAGGTTGTGATTGCGTCTACCGAAAACTCTCCCAGTCAAGTGGCGTGTATGATTCCGGGGGCAGCTGATTTTCCGTTAGATATTCGGATGTTTGATTATGAGGGACTCTGTAGGATTCCGAGATCCTCCTCAATTGTGTGCATTGGGTTCATGTTTTAGGCTTATGTCTGTGAGGCTCTGTGCTGGAACTGAACAGCAGTGACGGCTGTCGTCGCGACAGGTCTTTATAGACTTCCGATTGTCAGCGATCGATGATCCAGCTGAACCTTCTGCGTAGGCGTCGTGGTGGTAAGACGCGAGTGTTGACGAAGTCACTATGCAAGGGGTGCTTCGTAGGTGTGTTGTCACACACGACCAGAGAATCAGCGAATTCTTCTATGGTTTTCGTCAATTTCCGGACGTACGCGTCAACGGGGTTTGAGGTTTTTCCGTACGGAGGGCTGACGGAGGCAGATAGTGAGATAGTATCTGTGTAGACCTTGGCGTTGTTATTCCATGCATTGAAAATCTTCCGGCCAAGGGTGTTACCGTTGGAGCGGTAACCACCAAGATTAGAGGGCTTACCCTGGTGTTCGACGTAAAGCTTCTCGTGGGTATGAATAGGCCGCACGATACGTCTGATTCCTATTGTCGTTCAACAGATTACCACTAGTGGGTATGTTAGCGGGTGTGAAGTTGCCGCTAGCCAATTCTGGCCGTCCACCTGAATTTTCCACGTCAAAATGTTACCGTCGTTGGAGCGGTATCCACCAATATGAGTCGAGATGGAGTTTGAGTGACGTAATTGGGCCCGTGAATGGGACTCGACGTGAGGGGAGTCGTGCTATGAATGGGGCACGAGCTATGTGGCTCCCTTGAACGAACGTGATACTCCTTACACTAGATGCATGAGCGTACATCGCTGAACGAAGACTGGGAAGAAGCTAAGTCCCAGAAATCCTTGGGTAAGCGATGAAAGGAGTACTTCAGTCTCCTCGCTAGTAATCCTCGTTATCGGTTGTCCATTGATGAAAATGATGAATAATGGTGATCAGCCATTATGACGTCCAATATTGTGAAATACTAGTTTCTTACAAAACAGTTCTGAAGATCGGAGTGAATGTGTATGTTTATACAGTAACAACTCTGGATTTATTTCAACTTTTATTGGGGCTTTATTGCCAAGAAAACACAATTCAGGCCGGTATGTATCATCGGCTACATAGAGAGAATTAGAATGAGGGCAGCGAGTAATCATTAGATTGTTCTTATCCTTCTTCTGGAGCGTTGTTGTCCATGTGGCAGTGACAATTGTAAGCCGGATGTTGTTGCAGTGTGCTGAAGAGATGACTCCAGACGTGCGAGTACCAGTTTTGGACTTGGACCTCACTGCGGCGTCTCACGAAGAGCGAAAAGGTCGGGATAAACATCTGGTGGCCGGATGCGTTTCCAATTGCGATCTGCTGTGTGCAGCTGGATGCTGTGACGTTCACGATCATTTTTGCCTTTATGACTTTCCAGAGCCAGTGGGTGGCAGCCAGGAGAAAGCGATAGTATTGCTTGACGTCGTTAGGTTCG>XLOC_005681(mj441)GACCTCCACAAGCTTTTGCTTATCGTCTTGGCTCATACTGTAACAATGGAAAATGATGGATTGAAGTTTAGAAATCAGAAAGTCAGAAATTATGAGAGTTGTTTAGAGTTATAACTACAAAAAATAATGTTTAGATTATTTTTAAAAATTTAATTAAAAATAATTAAAGAATAATATTTAGAAGTAATAATGAGAATAAAATGAGAAGATGAAGCTAGAATGAAGCAAAGATGTGTTTCTCCTCTCGCCATCACCGGTCTTATAAGCGATTCGGGTCTGCAGCGGTCGCCTACCCTTTTTGCTCCTTTTTTTGTTGTTCTGTTGAATATCGCCTAAGCTTTTGCCCAAAATATTGTTCATTTTGAGGAGAGCGTCAGAGATACATCGCCTCTCTTCGACTTATCAGATACATGTCTCGGAGTGAAGCACCACCTACCCGACGCCCTTTCCCATGCCATGAGAGAAGAAGCTTGATCCGCTTGTAAGAAGAGGGAAAGAGAGAGAGGTAGTTGTAGGTGATATGCTGGTGATCTGATCATTACTTTTATGATTATGATACTATGAACATTATTTCAAAGTTATTTCAAAGTTGCAATGATTTAGTTCCTCATTTTTAAAATCAACTCGGAACAGTGAATTGAGGTGCTCAGTTTGTTTTGTAGATAAACGTAGAGGGGCAGTATTTCCTAAAAAGGAGTTTGATGTATAGTTTGCTTACAAGTATACTCACAAAGGCCTGACGTTAATACTAGGGTGTCACAAAATTGGCATTCAGCTCTGCTGTCTGTAATGTAAATTCTTGTTGTTTTTGTTTTTGTTTTTTTTTTTTGGTAATCAATGTTCTAGATTTTTTAGGGAATTCGTGTTTTTTTAAAACAAATCTGGAACTTTAATCGAATATTTTTAAATTGACAGAAATATTTGTTTTTAATTTAAATAAACTTTCAGATTATTTGTCTATTTCTAAGCACTCTCGAAATATTCAGAAGTCTATTAAAAATGTGTCAGGGCAATTCCATGAGCTTCGAAATCTGATTTTTGAAGAAATAACTCTGAAAGAATTTCAGATCTAAGTTCCAGAATGTAGGACCTCATCACTTGGAATTTTTTTCCAAAAATTTCGGTAAATTCTAAGATTTTTCGTTTACTGATAATATATTAAAAATTGTACTTTTCTTAAGAAAAATTTCAGCTTTTGAGCTTTTGGTATACTTATTCAACATTATTCTCCTTCCGCGTTCCAGTACTCTGACCAACTGATGGCGGCTGCGTCAGAACATCATTTTTCTTCTGTTCATACCTTGTCATAAACTTCTTCTGTCCCTTTAGCTTCATTCTTTTGATATCCCCTTTTTTTAGAACCTTTCTTCTCTTTATGTACACATACTTCACATTCTCATTATCAAATGATAACAGTCAGGTATAGATGATGAACGAATGAAAGTAGTGATACGCGTATTGATAATGTGAATTGGATATATATTCGGTTCACTGTGTTATTCACTTTTTGCCCATCTCCAAGGGCAGACTTTCGGCTGGCAATTGGGTGTGGCATGTGAACAACGTTTATGAAGACGTTCCAAAGATGCATGTGATTGTTCGAATCAGGTGGTTTGTTTGTGATTGTCACTGTCATTTGCTGGTAAGATTGATGGAGAAGCTGAAATAATGTGAGGTCCAACATTATTACAGTAATCATCAGGTTCAATAGATTAAATGCATTTTGATAGGTTTCGGGTCAAGCAATCTGATCAATGATCCATCATGTAGTTGCCAGTTACAGTAATCCTGAAAGTGCAAAATTATCCGGGGTGTATGTTCAATGTCGGCTTCACAACCACCTATCGTATTTCATCGTTTTGTATTGTACCCCATTGTTCAATTTTGTCAGCTTATATCTCACTTTTTCTATACAAAAATGTCCACTAACAAAATATTTGTTATTCCTTTTTCTATTTATTTGGATTGTGATCAATAAAATGTATTTGCAATATTTTCATGAAACCTGTTGAAAGAAGAAAAAACGTTCGAAGAAAATTAGAAGGAAGTTAAATCAAGTTAAAATTGTTAGAATGTGTCGATATACTTTCTAATTTTTCTGATCTAAAAACCCAATTCTTTGAAACTGCAAACAACCGGGGATGAAAAAAAAATCATCTACTAAAAAATTTATTTCATTGAAAATGATACTAGTTCTTATTCGGCGGCTTTCTCCACAACTCAATCAACTTTTTC>XLOC_045957(mj601)GTAACCATTTTTCATTTTGAAATTTTAAACAAAAACAACAACATTTGTTTGACCATCAGAAATTTCTGCAAAAAAAAACTGTAAAACGTTATTAAAAACACGAGTGACGTCACATTTTTTCTGGGAAAATTAACAAATTGTAGAAATACATACGACTAAATTCAGGTATTTCTTCATTTCATACTTTGAGTTACCGCGAAAGTCATGTTGAAATCTGAAAAGACTTTCTGAAAGTTTCTATCAAATTCAAGTTTGATGAAACTATACCATACCTAAACTTGCCGAAGAAAAATATTTTAAGATTTTTGTTTTTTTAATATTATTTTACAAGCCTTAATTTGGGTCACGAAGAAAACCTTAATTTGGGTCACGAAGACAAGGTCTGGAATTTTTTTGTTGTTGACAGTTTCGTATTCACTTGACCCCTCTTTCATATTCTCAAGTCCAAATATTTCGAGCAAGAACAAAAGTACCCCCAAACCGAGCAAACGAAAAAGTGCAATCAAGACGTGTTCAGGCCGGCTTATACCATCTTACACCGTTTTTAACACCCTTCCAAATCTGTGATAGCGACTTTTGCAACAATCAAACGGTATGTACTAGTGTCTGTTTCTTATCATTGACATTCACACCTTTCACCCCGGTT(ggag)CGAAGAACAACTATTTCAGTTGGAAGATGATGTCGAAGGAAGATATTCTTACCCGACAACTTATGTGTGGAGTTCTGCAAAGAAGCAATGCTTCGTAGTGCTCATTTTGATACAATTGGTGTGGTTGGTGTATGTCTAATTCCCAGTTGCTCAGTTGCTCAGAGAGTTTGCCTTCAAGGAAAAGCACTTTCAGAACATGCCCTTTTCTTGGCCGCCGTCGTTGCAAAGATCATTTTCTCATTCTTTCCCATATTTCTTTTGCTTGTTTCTCTAAAATATTTGGATTTTCCAATAAAGATGTCATCCGTTATTTGATTTCGTTTTTTGCACAGACAAGTGATAAGAAAAATTTATTAATGATAAGAAAAGCTAGAATTTTTAAATTTTCTAAACACTTTGTTTTTATTTTTTGGAAAAATTTGTTTTTTTTTCTGTTCCTGAATATCTACAAAATAAAAATGTGTCTAGTTGGCTCTATCCATGTATGTAGAAAAATAATTATAGGTAAAAATTTTGTTTTTAAAAAGAACTGAGCGAAGGAAATACATTTTTTTGAAGCCGTTTCAAGCTTGAAAAATTAAAAATTCAGTGATATTCCAAATTTACATACTTAGTCATCGGGAAGGAAATTACAATAAGTTGTTTTTTTAAATTTATTGCGCCTATTTAAATCAAATTTCTTTATATTGTGTTCAGTGGAAACCGCAATTTTCTCGGTGCTTCTGAGAGAATCATAATTGCATTAAACCTTATATAAATGAAACCACCAAGTTAATGAATTGAACCGGTTAAAAATTGAAATATATGAATCCAACATGCTTGTGGCGAAATCCAATAAAGTGTACGTAGTCGGCATCGTGGACAACCCTTCCCACTCCCAAACACCCCCTTCACGACGGAATACACCTAAAACCTGCAATAGAGAGAAAGAAACGAATAACACAAACGAAGATAACTCAAAAACTTATTTATTGTACAACCACGCCCTAGGGTCGCGCAGGCCATCAATAAATAAGAAAATAACAAGTAAAAAGTGGGTAATACATTAAAGAACAGAAAAGTAGGTGTTATAAAAAACTGTTCTTGTGGTCCGAAATCCTCATGGCGAAATGGAATACGATTTTGATCTTTAGACTATCAATTTGGTTCAAACTTTAATATTTAATTAATTCTAACAAATAATAATATTCTTGCTCGATTAAAAGGTATTTTCGAGTAGAATTAACCGTTGGGTGGCACGCATATCTTCGTGTACGAACAAACATAGCGCGCGCCGCCACCTCTCCCCCTTTCTTGGCGCGGTGCCAAGTAGTGGTGCCGCGTGGGTCTCGAGGGGAAGAGAGGTACGGTGGCGATCGGGCGCGTAGTTTGAATGCAACTAGCTCGTTTGGTGATTATGTAAGTTGAGTGTATCTCGGCTTATACTTAACGGTTTTGAATGAAATAAAAGGAATTAACAAGAGAGTAATTAGGAGAATCTGGCAGTGTTAACATAGTTTAAGAATAGTGGGATATTCCAATGAGAACTATGTTATAGCGCTGCAAGGTCGTCGATTGTAGCCTTTTTAGGGTGGATTCGACGACAATGCTTTCCAGACATTTTTCAATTTCTATTTCAGCAGCCCTCACATCTCATAAACGGCAGATGAAAATGAAATGCATTCGAATATTCTGACGGACACGGAGCGCGGGAGGCTTTTGAAGCGGTGTGAGGCAGACAGAGAGATTGCATCACATTTGGAGGAGTATGAGCAACAACACAAAGGGGGGGGGGGGGTCTGCCGCTCTTTCTACGAATGACCACCTCGTTGGTCTGCTTTTCTCCCCACTAGTTTGCATACATTAAATTTCAATTTAGAAAGCGTCAAGCTTAAAGCTTTTAAGAAACATTTTGGAATGTTAAGAATTGAATTTTTGTGTTCTTGTCTGTATTGACTTTGCTGTTTTAGAATGATTATTGTTTTTAAGTTGTTGAGCATGAAAAATGAAACGGCATACAAAAAAAATATGAGATTAAGAAAATAAATTGTTGTCACAAGAAAATATAACAAATTTTTAAAGGTTGAGTATTTACAAGTTGTTTCAAAGTTTCCAATAATGTTTCTTCAACCCCAACATAGCCAATACTTTTTTAAATGTTTTTTGCAGATCTAAAATGTTCTAGCTTCTGTTTAGTGTTTCCCTCAACTTCCTTTGTAGTTCATCTTTCTTTTTTCTTCAAGCTTTTTTCTTTTTTTTTTCAATTTTTTTTGCTACATTTCTGCTGCAAAAGATCTGGAAATGCGATAAAAAAACTAATTCTTCAAGTGTTTTTGTTGAGAAATGTGAGTAGTATACAATAAGGAAAACAAAAACAACCCAACCAGCTTTTTCCTATTTTATTTTATTTTCACGTATTTGGTCTATGAATTTCGGTTTTTCTATCAAGTGTAAAATCTGAGTTAATGAGGTTAGGGAACAATTAAAACATGGTTCAATGAGTTATGTCATGTAAAGCTAAATTTAGAAATTGTCGTCTATTGTACTTGTATTAGTGTTGTAAAAATTAAATGTATGTAAAGTTTTCAAAATGATAAAGATATCCAAATGTGAAATGATACAATCATCGGTCAGAAACTCACATTGTTCCTGGAAGATTTTTGAAATGTTTTCAAACGTTTAATTGCACATTACAAACGATCGCATCATTTGTAAACAATTTCTCAGTTTCAAAACGCTCTGAACATTGGTGATTGATATTTATGAAGTACTATAAAATTGAAATTTATCTATTAGTATCCCCGAGCGTTCCTTAAAGCACTGCATACCTAAGTTTATTGATTTCCTGCTAGAGTAGCGAATTTTTTTCTATTGCTCTATTATAGTCACCAAAAACAATTGATAAGCTGCTTCAAATTGACGTAAAAAATGCCTTTTTTGTAAAACTCAAATTATGTGACCAAAAAAACGTGAAAATTGTCTCCAATTTCAGTATTGCACTAAATATGAACATGCGATAATAAAATAACATGCGATAATTTTGCATGCGATAATAAAATAACAAGACGCGGTATTTTTTGTTTTTTTTTACCTCAACTGAAAAATAAACCAATTAATTAATCAACTTAAGTAACTAATTGGAGAAATTAACAGAGTACAATATTGTGCCAAACTGAAAAAGTATAGCAACTCTTTGCTCTAGCCCTCCAATGCAATAATTTTTTCTCCATTTGATCGAAAGTTCCCAAGCGCATTAGTTTCTGAATGTTGACTTCGCTTTTGTTTTTCTCTTTCCATTCTTTGTTCAATTCCTCTGGATTAATCCAATCAACAATTGTGTTGTTTTGTAACCCGACACGTTTTAACTCTGCCGTTTCCTTCCTTGTAACCCGATACTCAATATATAACACTTTTTTGAAACTCTTTCTCTCGTTCATCAACTGACCTGTACAATGTGTTGTCCGTCACTTTTTGATCAGTTCCTTGAAGACCTTGAAATATGCAAGGCTTGTAAACAATAATGGTGTGCAGATGTTTTTCTGTGTATTCGTATTCTCATTCCGTGATGCATCCTTCTGCTCGATTGTGAAAAGAGGATAAGAAAAGATAAACAATTAGGAAGAACGGTCATCCCCCGCTTACTTGAACTTTGTTTATTTGCTTTACAATTTATGACAAGTGATTCCTTTCCTTAAATAGCATTAAAATGAGCAAGATTCTCGTAGAATTGTGATCATTGTCATATGTCTAAGACTTGTTCTCCTTGAACTATTTGAACTCTAGTTTTACCGTACTCCTTCTATTATCATAGCCTCCGTAGTAATATTGCGCTTGCCGCGCTACTCTGAAAAAGTATTGCTCCCTTTTTAATATAACATATAGTGTAACTTTGAATCGAAATCATGTTCCTTGTCATTTTTTCCATATGACTTTCCTAGTTTGGAATATTACTGAAAAGATAAAACATCTTGAAACTGAAAAATATCATTTTCAAAAGTGCAACTTAAATGCTAATTCGAAAATAGCTAAAATTATTCGAAAAATGCATTTATTGCCTACTACGCCTGAACTTTGTTGCATTGATCCTTCCTTTCAAAAGTTGTAGTGGAAGTTTTGGTAAATTGCTAAATTTTCAACAAAAAAAATTTTGACAGTGAAGAAAGAGATTTTTTTCGGCACTTTGAAGGCTCATAACTTCCGTTAGAGAAATTTTTGAGATGTTTCGCTTTATCATATCGACTTGTACGAGCATAGAGAATGTTTTTAAGCATAGTCTCAAATTCGACTACACCACCTTTAAGCTTCTTCCATCCTGTCGTATTGAAGATTTCAACTAAAATTAGGTCATTTTTAACCTATATTTTTTTAAATTTTGAATTCACAGCCATATATATTAAAAGTCTGATTTTTCAAAATTTAATGTACTTACATACTAACAAATCTTTTTTCCACTAACCTACATACATTTCACTTATGATCTTTGTATATCGTTCTACGCTGACTCATGAAATGTCAACTCAACTATGGTTTTGAGTACCAACACACCAACACACCTAAACACTTCGGTAAAGTGATGGGGGGTCGGCAGAGTGAGAAATGGAAAACGTTGAGAGTTTGGTAAGTACCTTGTCTTGCGAGCGAACAACTGTTCGGCGTAAGTTATGTATATTCGTCGGTGTTCACAATTTCAAATCATACACCCACCTACCCACTATGATCACTTGATAACGGAGGACACACTCACGCCTCGAGGGGTCCATTCAAGCAACGTGACTTGTTCACATAGACTTATCATCTTTTGACTTTTTTCTCGCTCACGTTTCCCACCTCAATCAATCAACGAACAGCGTCGGCGTGGTTTTCAATTTCATTATCAATTTTATCATATTTTTCTTTCTTCTGTTTCATCTATTGCTGTTTCTGATTCTTTTTTTTTCTGATTTTGATTCAATACAAAACAAACAGACCTTTACATCGCTCCACGTGGAACG>XLOC_041869(mj437)TCAATTGCCATTGGACAAGAAGCCCACCGCTACCGCATCTACCAATTGATACACACCTCAAGGCTTTCGGATTAATCCGCCTTTCTATTTCTCGCTGTCTCATTACTAAATCATCATAATACGCATTCATGTAGAATAGTTGACCAACGGGATGCTCATTTCTAATAAAATTGTTTCCCATCTTTATTTCCTGAATTTCTAATATGCACTTTCAAATTATATTTTTTCGTTTTTTGTAATGAATTATTATATTATAATACTGAAGTTTGAATATTTGACACTTCCGTCATAATGATTCTCAAAAGTACAATGTCTTCATGGTTAAAAGTGTTTAGTTAGCTTTGAAATTCACTTTTAAACATTTAAATATCACTTGCTAATCAATTTTTGTTACGTAAATATAGTGTAATAAGCAAGACTAATTTTCAATTGGCCCGTTGGGGTGCAAGACTATTAGAGGCTGCAACACTATTTCTCTAAAACACCATAACTTCGGAAACGTGACCAACTTTTGTGGAAAACTCAACTTCATTCTGTTTAAATGATGAAAAATATATACTTTTCCAATATTTCATTACCGTATTATTAGTCTTGAATTAGCTAATTTATCTCGCAATCTCTATTAGTCTTGCACGCCTACAGACCAATTGATTGCATGCAAGACTAATAAAGGAAATACGGTAATTAGTTTAAGTTTAATTTATCCAAAATTGGTTATTTAGTCTGAAATTATAATAAAGTATTGTTGGCCTGACAAAAAAAAGTGTAAGACTGTTATACTAATATTAAAAGGCTGGAAGACATATTTTTGATGATAATTTTTTGAGACTGATTTTTTGATTTGATTGACTTGTAGCGGTGATGAAATAATATGAATGAAAGTCTAACAGAGGAAATACGGCGTATGCTTTTACAATCAACATTTTACTACATTTTTACTACAAAAACCTTACTATTGTATAATGAGAAAACATTTGATATTAGCGCCAAACTCTTCAAAAAATGCTTATCTAACTAAAAATTCTAAACGACGCAACTGAGATTTTCGTGGAATATATTGAATAGCAAAACTAAATCAGTCAAATAGGCACAATTGAGTTTTGTTACAGAGACAATATTTTATTGAATTTCCAATTGGCTGATGAACTTAAATTACCGCATACTAGCGATGTCTTAGAGAAAAATAATGTTAATCACATTTTTCCGTCCGCGTTTCTTTGTTCATTTCTTTTCCAGATTGTTCAGACCTCAAAATGAAGAAATATTCTTTCCCAATACACACTTTCGATCTTGCCTTAACATTATATATTCTTCTTTTCCGCCTTATAAAACTTTATTGTTGCTGAAGAAGATGATGGTTTCCGACAACATAGCAGCAACAGCACACACACACATATCAATATCATTCCGAATATATACTTCTTTTCATGTCATGCATTCAAAATTTTTAAAATTTCTTTTTTTCTGGCAGACATCCAATTCTAACCAAGGCGTCATCGTTGTCGCGGTCGGTTGGTCAGGCCGTTTTGCGCATGTGCTGTCATTTTTGTTAGAGTTCTGTGTTTTCTCTTCGTAAGTCAGTCATCCCATTTCAACTCTTTTATCTTTCACAAATTTTGTCATCCGGTGCAGGGCTCTGTCGTTCGAAGAATTATTGGAGCATTTCCATTTTAAAGGCAAGTGGTTATTCTTCAACTTCAATTTGGTTCTAACCTAATTATCCCAGTTTTTGTGCAACGTTGTCAACCAAAATTAGTCGACTTGCACTCACGACAGCTATTGGCTCTTGGAAGGAAAAAGAAGACGACGTCGTTTTCAAAGTCATAAGCGTTACTCGTTTACGCGTTTCCTTGTTCTTCCCTTTCTTTTTCTCTTTCCCTTTCTCCAGCATAAACTAGCGTGATAAAATTAAAAGAGCTGCTATGAAGCCTTCACTCAAATTTTTCATGGATAACAATTGGGTGGAGGAACTTTTTGAGAAGCTCAAATCAAGAAATATGTAGAAAAGATACGCATGAACAGCTGAAGTGATTTTCAATATCTACTGTTACTTTACCAGTATTGAAACTACATATTCATATGAAAAGCTTGCCACTCAAGATTTTTAGGGGATACGAAAGTTGATTATTGTCTCAAAAACTTTAAAACATTTTTAAAAACATTTTTAAAATGCATGATGCCTCAGCAGTGATAACCAACAAAGAAATTGGGCAAAAAATGATCACTTTCGGGTTTCTCTTCTCACTTCTTCGCATCATGCTATCTATCTATCAACGCTTATCATTGTCGTGTCGGGAACGAACAGAGGGACCATCGATAGGTACACACACACACTGAGCCCATTGCTAGGAATCCACGGCCTCTTTTTTCATACTACTCCCCGGGCGCCGCCACCCGTCAAATTT>XLOC_040158(mj602)TGTCGGTTCCAGACATTTTTGAGCATTTAGTTGTTGGTTGTGTTTCTGGAAAATCTGCAGGTTAGAATTTTCGAGAGGGAAAAAGTTGGAGAAAAAAATTGCAGAGAAATTGAAGATTTTAGGAACACAATTCCACATCAGATTTGTTTTCAATATGGTCGGGATGAGTACGTTTTCAGAATTTAGAAATTTAATATAGTCTGTAAATTTTATTTTTCACCCGAAATCTTCGCACTTATCTCTGCAAATCACAACTGTTCCAAAGATATTGCTCAAAAAGTATTCATTTTGAAAAAACATTTCCTTCGAAAATTCAAAAAAACGATTCAAAACTACCGCTCAATAAAAAAACAAGGAGACATGAATACGCCGCTAAATAGTTGTGTGTGTAACCATAAATCGATTATTTTTGTCTAATCGAGACTGTCTTATCGGGGCACACAAACACCCAAGGACAAACAGGCACAATTAGAGATAATAATTAATGAGCACAATGTAGTAGATGTCTGATAGAAGTCACAAACTAGAATAGCAAAAAGTACACGGTCATGCAAATGCGGTATTTTAACGTGAATTTTTATATGGAGTATTATCTGTGAGTTTTTTGTGTTGAAAACAACAAATTGTTGTTAACTTTTTGATGTGTTCATTTCTGTTTCTTTGGGTCATAATAACTTTTTCTAATGTTTCTATAATAAATATGCTATGAAAATATAAATGTTGTATTCGTATAAAAAAATATAAGCACAATTTTGAAAGATGAATATTAGAATTAAATATTAAAAACTGAAAGCTCACTTTTCAGACAATTGTTGAGACGCTTTGGGTCTTTACGGAACTTGGTCTTTTTACAGACACAACAGCTGTAAACAAAAACAATAGAAATAAGCCAAATGATTATTACAGTAATTATCTAGAATATTGTTTGGAAGATTGAATGGAGCTGTGCAGAAAATTGGGATATGGAATAAGTTGCGAAATTGGCAAGCAGCACAAAATTCCTACTGTGGAATGCATTATCTGGAGATTCAAATCGTTTATCTTCACATCAACACCTTCCATATTTTTTCTGAACTTCTCCATTTTAGCGTGATTCATGTGTGTGCTCTACTTAAACAATAAATTTTCCAGACTTGGGCACAGTGCGAATTGTAGTTTTTCTGAATTCCTCCATTTCAACGTGATGTAAAGTGGATACGAATGTGGAGTCTCCTATTTTCTTAGTAAAAACCTTGTCCGGGGTGTATACTTCTTTTACTTCAGAATTCTTAAATGAATTCGATGTGCAGTTTCCACGAGACGTATAGCAGCTGTAGTGTCCTCCAACTTTTGGCATTTTCCGGCAGATATAGTGTCATTCAAAGTGAACACACCAAGTCTCAATACCAAAAGAGCACTCCATTGTGGGTGAATGAGCCGACCGGTGCAACCTAAAAAATGTGTGATTGAAATCTTATCAGATACATATGTTAACTTACGAGCTCCACTTTAGCAACAAGACGAGGAGGGTTTTCTTTTCCGCAAATTTTCACGTTGTAGTGAACTAGACATCTTCCACACAAGAGTGTATAGTCCAACAATGGAAGGATGTCGGTTCCAAACTGATACTGATATCCAAGATCGTTTAACAGTGCCTACAACTTCTCATATCAGGGGGCAGGATAGCTCAGTCGGTAGTGATGACCACTAGTAATCTGGCGGTCACGAGTGCAAGTCCTAGGTTCACCAGCCTCTATTGGGAAGTGGAGCAATCCACGACTGGATTATCGGCCACAGTCCCCGGCTAGGACGTGGCTTAAATTATAACCCAGTGGGAGCACCACCAGGCAGTGTGCCTGACTCCAAGATCTGCGGTGCATAGCCCATTAAGAACGGATCGTCCTTTAGTCTTTTAAAATATTCTCCATTCCCATAACAATCTTGAATTTATTGTAGTGTCCTGTGATTCTTGTTGATCCAATCACAATTGTCATTGTGCGCTCTACTTGAACAATAATTTTTCCAGAGTTGGGCACATTGCGAATTGTAATTTTTCTCAACTTCTCCATTTCAGCGTGATTCATGTGGTGCATTTGTTTTTCAAAGTCGACTGGAACAATCTTGAATTTATCGTGGTGTCCTGTGATTCTTGTTGATCTAATCACAATTGTCATTGTGTGTTCTACTTGAACAATACATTTTTTTAGTGTTGCAACAATCGTCGCGTCTACCAACTTCTCAGTGAACACCTTGTCCGAGTTGTAGACATTTTTCACTCCACAGTTTTTGTAGGATTTTAATGTAAAGTTTCCACGTGTCATACACATCAGTTTTGTGTATTTAAGCTTATTACAAAACAGATGTGAACAAACGAGTTCAACAAAAAAACTCATTGCTATTCATGAAGTCGTTGAGATCAAGCAACTGGCTGAAATACTTGAAATTAAATTTTAAGTTTCATTTAACAAAATACATACTTTTCCGTAGTGTTCATTCCCGATTGTAGATCTTTAACACCCCATCGCAGATCATTTATTTGTTTGTTAACAATCTCGGTGTAATGAGAGGTGGCGCCATGATCGTATACAGCGCCGGCCGAAAAAACTGAATAATTTCATCTCTTATTAAACCCCTGCTGCTCATACCTGAAAAACAAATGGCATATTTTTCGGATTTTTTTTACAACAAAAAACTTACTTTTGCATTCGGATTGCAGCAACTGGGTGAAGGAGTGGAATGGATTTTGATTGAAGATTAATTTTTCCGGAACGCATCTCTGTCGTTGTGCTGCTTCTTTTTCAACGTATCCAACACCAAGTCGAACAGATTTGAGCTCTCTTTGCGTGAAGGGAGCAAGAATCGAAAGAAGTCAAAGAATAAGAATAAACAGTTTCATTCTGAAAATAAATGGAAACAGAAAGTATGTAGAATATACAAAGTCGAACTGAATATAGAATGATGATGTACATGTTCTCCAGGAAAAAATGATGACAACTCCGACGCAGTTTTTAACCGCGTTTCTCAAATCTTGCGTTAATCAATATTTTTCATAATCATTATTGATTGATTTTTTGTCATCTAAGTCATCTCCTTGATTTACGGCCCAGAGTCCATACCTATTGTTATTTGATCCCTTGTGATTTTCTAATTCCTGTTTTGACTTCTATTCTCCCAACTTCATATGTTGTGTTGCTACTTTTTTCTTTACAATTTGCCTTTTTTTGGATAAGCCGCAGCTTGGCCTGCACAATGGAACAAAATTCAGCATACTCCGAAATTTTTGTAAATGACGCACACCTGCGAAAAGTACGCGAACAATTGGAAAAAAGACAAAGCGAGTTCAATTCGAGTAAACATGTAAGGCAAAATTGAAAACGGGAATATTTTAAAACGTGTTAAGATGATGCTGAAATGATTATTAAAATATAACATTTCAAAAATGTTCGGAAAATTTTTAGGTAATGAGCAATGAAAACTGAGCTTGGTCAAGACGTGTAAAGCACTGTTGAAGCACTGACCCCTACAATACTGAAGTTTTCATGTTTTGGACTATATTACAGCGTATAACTGGAAGATATAATTTTTTTTGATGGACGACTTTCAAAAAATTAATAATTTCCCCACTGGCCGTAGATCACGAAAAAGTTGCGGTCAAAAATTGACCAAAACCTGTCAAAACAAACTTGGAAAACATAGTAGCCGCCAAATTTCAAGAATTCAAAAATAAAATTAAAATTAAAAAAAAATTTCAGAAACGATACAAGATATTTAATAAATTCAAAAATAATTTATTACCATAAAATAAATAAAATAAAATCACATGAAAACTTGAAACATTAAATTCAAAATCTGCTATTGTACAAACAAAAAATCCTTGATTTTATATTGATGGCGATCACCATATGGTTTACCTGAAAATAGAACAATGATGTCACTGGCCCCGTGTAAAGTATTGAGGTTTTCGTCGTGTTGTGCCCGAAGAAAATTGGATGGATCTCGGTGGTTGGTCGGTTAATTTGACAACATCTTTTTTTCTGTTTAGGTTCCAAAGTTTTTTTTTCTCAAGGAATGAGTCATTTTATTTGAAAATCACGATGTTTTTTTCTTTCAGAAAAGTCGCGGTAACTGATTTTTTTTTAAATGAAAATTTGTTTTTTTGTTGCTACCAAATTCACATGCATTATCATATCATTGTTCAATTGCATTTAATCAAAATTGAATAAAAACTAATCTGAATAGAATTGTACTTCTATTCACTGGAAAATGAAAAATTGAAGAGATCTTGTGATTCCAGCTGCTGATTATTTCTTTTTATTTTTACACTTTTGTTTGGTTGTGCTGGCATCCAAGCATATTCAGCTTCCCTTTTTTTTCAAACTTTCCAATATATTGTTGTTACCTACTCAAGCCTAAAAATCAGAGACGAGGGCCGTAGGCCCTCTGACTCTTAAAAAAAGTTCTCAAAAAATTATCAAAAACTTCAAGGGGCCGTAACTCTTCAGTTTTGTTCGTAGCGACCCCATTTTTTTCGGGCCCCCAGGCCCGCTAGTCAATTCGCATATTAATTTTGGTCAGTGCGTAAAACATTTTCTGTCAGAAAATATAGTTTTCGGTCAGTGAATAAGCATTTTTTGAGCTGTGCATTAAATTTTTTGGTCAGCGTATAGAAACTTTTTGTCGGTGCATGGGAACTATACATATTTGGTAGTGCTTAAACAATTTGGTCAGTGCGAGCAAAGTCAGAACAGGTTTGAACTTTTTTGTAATTTTTTTTCATGGAAAAATTAACTAAACATTATTATTTTAAGCTAGACTTTCCCACATAAATTTTTCAAAATTTTGATTTTAAGTAAAATTTAGTGTATTTATCACCCCGGTTTTCTAAGCTGCTTATTGGTAGACGCCTGTGAAAAAAAAGCTTTTGAAAACATGTGATATCACCACGCCAAACAAGTTGAAATATATGTTATTTGTGAAAAAAACACTGATCAAAACTCAAAAAATTTCAGCGTCATCCCACCTGTCTGTGTCAAAATAACTTTTGGCACTGATTAATTATCATCTGTCAGTCTCTGCGTCTCTCAATCCATTCTTCTTCACACCTCGAAATCTCACTCTCTCATTATTCTTGTCGAGCCGCGGAGCCTCTCGCCGTTTTTTTCCGTTTGTCCCCCCGCGCTGCCGTGTCTCATTGCTTGGGCTCCTGAGTTGCTCCGTGAAAATGAGAGATAGAGAAAGGAGGCAGATATACTCTATCGAATAAAATCTGAATGATGTCGAGAGAAACTTGTGGGAGACAGGAGCCAAGGGGGGGGGGTCTGGGGATTGGCAAAAAGTTGTGTTCTACGCTGCGCAGGTTTCCAGCTCTTCCTCTCATTGATCTTAGTTTCAAGTTTGCACATGAAAAGTACATTCCTTTGTTGATGTACTTTTTTGCTATTTATCTTTCTGTAAATGTTTTTCTAAGTATTCAGTCAAAGTTTTGCACCTCTCGTGGCCTTGTTGGTGGAATTTAAATTTTTTCATGTTCCTCGAATTTAATAAAGTACTAGTTTGAAAATAGTTTTCTTTGTGAAAAAGTGCAAAAGTGGAAATAGTTTATTTTTATGAAGATCGAGAAAAGAAAAATGCTTGATTTTCGCGTACTATCCAATTATTTTTCTATTTATTTTAATGAATCCCAGCTCCTTGAAACTTTTTTTTCATAAACTCAAATGTCCCAAGCCGCGCATTTCTGAATGGAACAATTAGTGATATTATGGTCCGACATCTACCGAGTTCTGCGCTTTTTTTAGCAGTGACCAACATCAATTACAAACATCTTGTTTTTGACATCACGTTTTTCTGCAATAGATTACAATTATCGGAATTTTCCAAAACTTCAAAAAATGTTTTTTTTTCTATTTTAATTTTCCTGATCTTATTTGGTTTCTTATTCCTAATATATTTTCTCTCACCAAAGTAATGTTCCGATTCTCGAATTAAGAAAAGGGTCCGAATTTCCGCCTGACTAACTACATTTAGTAGTTCCGTTAGGGCGGGTGTCTAGAAAAAGTTTGGTAGAAAACAAATGTTTTTTGAATTTAAGATTGCCAAAGTAATAGACAAAAAATTAGGGGTTCACTAAAGAGTATTGTTTGGTTTTTTCAAAAGTTTTTTTTGAACATTTACATATAAGAAACATTTTTCATTAGTGTTGGCAGTGCTAACACATTTTGAAAATTGTCAATTGCCTAAAATTTTTGGTTTCCACGCACCTTCTCGTTGCAAATAAATATTTAACTATTGTCAAATATCATGTTTCTTTATTTTGTTGCTCATTTCTCCTTGCTTCCTTGATTTTTTCTGTTACTTCAGTTATTCAAATCGCAACTTTATCAAAATATATATAATACAAGCTTTTTGCATAAAATTGCCTGAAAAACGTAACTTAGCAGTTAACATTTAGGTGCAATTATTGAAAAAGTAACGAAAATTGTTGATGCACCATGATTCTTTTTTTTTAATTTTACAGAATACTGAAAAATCTGAAAATACTACGCTTTTTCGACTCGACCATTCCAATTTTTACTGAAATGACAACTTGAAATTTTATATTCAAACTAGAGCATGATACAGGAAGGAAAACTGGCAGCAGCCAAAAAATTATTTAAAAATATAAGCTTCTGATTTTTTATTATTTCGAAGCAAGTGGGTCAGCTTTAAAATACTGAAACTATCCCAAAGTTTTGGAATACCTTACAAATCTGAAATTGTTATACGATATTTTCTTGTTATTTTTGTTGATTATAATTTTCCAGACCTATGGTTTATCTTGAGCAATCCAAAGCAGGAGAATGTGTGATTCAAATATCGCCAGAAAGTATCTATTTTGCACTTTCATTCATCCCTCAACACTTTGACCTCCCTTCAGGTCAAATTGCGTTTATTTTCATTTTGAAGACATTGAAACCTAGGCTAAACTCTCTCAGGGGAGCATTTCCCATGTCTTCCTCCTTTCCGACATCTGTTTCAACCCGACTTCAAAATGAGAACCAGACTTTTTACACGTCATTCCCTCTCATTTTCTTTCCCTCTTATCCCAATAGTTTTTTGTTTCCGGATGTGAATAATTTATTAATTTTTTTATGTAGCAATAATTTTGTGCGGAAAAATTGATCAATTTTTGGCAAAGTTGGTGAGGACATTTTTTTCATGATGTATAAGAAATTTTCTCTGTAACCATAAATAATACTTTTAAATTTTCAAATTTTAAGCATACATTCAATGTATGTTAAATTAACAAGCTTCAAATTGTTTTGTCGAACATTTTTTTTCAATTTTGATTTTAAAATTGAACTTTAAGAATTCCCCCTTTTTCCAGTTTGTAAAATTCATTGCACTTTAGGAAACAAGCAAAAACTGAAATTAGTTTGTAGCAAACGTTTTGTCCCTAAAATATATCCCTCACAAAACTGAAAACTGAAAAAAAAAAGTTACTAAGGATTTTATTTGTTGGTCAGTGCAAAAGTTCCTTGGTCAGTGCAACAGTTTCTTGGTCAGTGCAAAAAAGCGCATATTCTGTAAGATCATAAAGTGTTCGGTCAGTGCATAAACATTCTTTGAGGTGTGCAGAACGTTTTTAGGTCAGTGTATAGAAACTTTTGGTTGGTGGTGCATGGAATTTTTTTTTGTCAGTGAACAATTGGTCAGTGCGCGTAAAATCAAAACACGGCTAATCAAAAAAAGTTCTGCTACTTTAACAAGCGCTGTAAAATTTTTGGTCAGTGCAAATGACATTCTGGTCAGTGCCTTATAGACTTATAAAACAATAAGTTATAGAGTTGTAATATCTTTTTTTTCTCAACTTTCAATTTTCCTATCTACCCATCTCACTTTTTGGTTGTAATTCTCAATAATAAATGTTCATCCTCACAACCAATTCATCATTATATAAATAGATTGGCAACAAGAAGACAACAACGTGACCAAGTGTTAATGAAAAAATAAAAAAGAAGAAGAAGCAACAACTGTTCGGATATAAGATAAGCAAACGAATCAATCACAGTGTGAAAAGGAGCGGGATCGGGGAATATGGGAAAATCGCAAAGCA>XLOC_008459(mj603)GCGAGCTTCCAACAACTTCCCCAAAGGACTATAGGCACATAAAAAGCAACACACACAAAATAAATGTAGTCGTTGAGCAAAGCGACAGAGAAATGGAAAAAAGGCTTCAACGCGTTTTTGTCGTTGAGATTTGCGATGGCGATAAAAAAAATGAAATAGAGTGTGAATTTGAGAGTGTCTACTACCACCCACTTTTTTCATATTTTCCCCTTTTGGTGTGCAATGTTTCTTCCAATTACTAAGTGAACCTATTTCAAAGATTAATTGAAACACCAAGGTTTTTTAATGCTAACTTTTTTTACTGAAAATGCTGATAATTACTGGTAAGCATGTACTCAGCCATGTGCTCAGTCATGGTGGCGTTAGATGTTTCTTTCCAGATTCCCTGAGATTTCAATTTTACGAACATTACATTCGGCAATTTCTACATAGGTAAAATTGAATCTTATCAAATTACAGGGAAAAACAATTTACTTTCTCGTGATTTTATCACGCAGCCATGAAACCCCTGTTTTCTCAGAACACATTCAATAAAATGTAATATTTTATTATATTATTAGTTCTAGAGAATGTAATAAATATCATTGATAACCTTTTACGTATATTATGTTTTTATTGAGAATAAATATTGTATCTGGTTTTTTTCTGTCATTCTCTATAAGGAACTCTTTCAGAAAGGCAGGAAGATTTCATAATACAACTTTTTTTCCTGATTTACTGTTTACATTGCTTTAGATATTTTGTCAAAAGATTACATTTTTTCATTAAAATTTATCGTGTTTTATTTTTCTAATTTTCTGTTATTGCAGACTGCGTAGAATATTTAGCAGTTCTAAGAAATCGTGCTTATTGCAAATACTCTGAAGCAATTTCACTAGTGTTGAGAATTGTGGGTTTTTTTTTAAATTGAAATGTTGGTGTGAACTTTATTCTAAGCTGTTCTCCAATTATCGAAAATTCAGATAAGCTGAAAATTTATTTTATTTTTCTAAATTTGACTCGGCTTAAAATTCGTTCCTATTTCATAGACTCATCTTACTGTACATGATTCAACTTCTACAAATGAAATCTCAACTTTAAAATTAACCCCCCTGAGAGCATAGCATTCGTTTAATCTTTCTACTTCGACTAAATGAAACTTCTAATGTTGATCTTTTTTCCTGCTTTCTCTTTTATTCAGTCACTCTCATTTCTCATTCAATCAAAGACTTTACGGAATTTTCTCACGGTCAGAAAGACTCCAAATGCTGACGTTTTCTATTTTCCAGACGAACAAAACCATCAATGGACTCAGGTGTGTTTTGTTGAGGCCGCCTCATCCCATGGGATTTTCTGTCGATGTCAACACAGAGTGAGACGAGAATAAAAAAAAGAGAACTTGCAAAAAGTTCATCTCCTTTAAACTTTCTAAGATTACATTGTTCCTTGTTCCTATTGTTCCTATTCCATGCTTTTGAAGCCACCAGGATCTATTTCCTTTGACGCTTCATTCAATTCCTTCGAGAACACTTTTAAACTACCACCATTTTCAGGCTCCATACTATCATCGCGTACCCATTATTTCTTCGGTTTTTTGTGTTGTTTTCTTTTGTCCTCTTTTGTTCCTTTCCGTTGGCCGTTGTCGTTTAGTCAATTGACTTGTTCAGCTGGTTCTTTCAAAACGAAGAAAAAACATGCATGATATGCGCTATGATTGATGAAATACTTGACACGGCTGCTCGTTTTTTTGGAGAACGAACATCGACTAAAAAGAACAAAAAAATGACTCGTATTATTGATAGATACGTTACAACTAAAGCACGAGTAAACAGCTTTCGCCTGCATGGGTTTAAATTTTTTGATTCTGAAAATTTTCTCTAGAAAACTCTATTAAATCCCCCACATTTTGTTTTAAGACTTTATAATGCTCGATGATTCTCAAGACAACTCACAATCAGAATCACATCATATTTTGTTCTCAGAATATAATTCCAGTGCTTTGAGACAAGACAATTTCCAGTTTGAATCCTTATAATCTATTTGAAATTGATATTCAAGCGTCCAGTATAGTCTATTGCAATGTTCATTGAGCTTCCCCTCTAATCTAGTTATATTAAATAAATTAGTTTATCGAAGGACTATGATTTAAATTAAAAATAATTTTGCGAGAGAATGTTTTAAAAATGTTTTGTACTTTCAGAAGTTTGCTTTTTAATTTTTGATTTTGAAGACTTTTTTAGAAGTTGTAAATTACAGCATTGAAAGGGAACTGAAAGCACCACATTTTTAAAACTTTAAAACTTTTTCGTGTGAATTTTGATACACCTCTTAATAGAAGGTAACTTTCTAATAGTAATTCGAAATATAATTTCAATGTTTTAATTTTTGCATTAAATGCAATTGCGTCACAACAGTTATTCGAAAATTTATAGTAACAGAGCAAAAAATTTTATTAAAAAACATATTATCAACGCCTACTTGCTCACGTGAACCTGAATCCAAAGTTGATTTTTGAACACATTCGTCACCAAAGAACTGACCTTTTCATCCAAAAGTTGCCAATAATTCTTCTTCTTTGTTACAACTTTGCTCATTTCCATCCTTCTCGTTTTCTCTATCCTTTTAAAGCCTCGTGTGGCACCATTCATGGATGGTTTATGGCAATTATCCAGCCACAAAAACGACATTACTTCCGAGAGCACTCTCCTCTTTTTTTCCAACTCACCTCGGCTTCTGCTCTTCTTCTATTTCATTCCATTGCTTCCTCGTTTTCAGACGTCTTCATCTTTTTTTTTTCTTTCCACCGAACCAAGCGCGTTAATTAAACGCCTCGACAATCGACGCGACTCTGCGAAAAATGACGACGTCAAAATAACATCTCATTTTCCTTCATTTTTCTTTCATGATGCTGACGTGGCACACTCTCTGCTCTCGCAGTCGCTTGTATTGGCGTCAAGTTA>XLOC_010885(mj440)ACGAGAGAAGCGATAAGGCTACTATCGAAGTTACTACAGAAGAAGATACTACTATCGTAGATATTATAACTACTACAGTAATTATTATTATTATAACAATTACTATTACTACCACGGTTAATTTTTGTTATTTTAATTTTAATTTTTTAAAATAAAGTTGCTCAATGTTGTCAAAAATTTTATTTTCAAAAAAAAATGTTTTAATTTTTTTTTGAAAAATCCATAACTACGCCAGTTTTCAAGATTTTTACTTGATTATGCACTTAAAATGTAGATAAATATTACAATAACCAAAAAAAAATTCAGTTTAATTTCTGCTCATCAAACTACAGTAGGCAAAGGCTTTCGCGGCGGATCCTGAAGTTTTCAAAATCGTCGTAGTCCAATCATGCTTGGCGTTGTTGGATAGGTCTCGGTGTGCTGGAAATGAATTTTAAAATAAAAACCAACAAATTTCAAATTTGCAATTTTAAATTAAATTTTTCAGATGGAAAACTAGATTTTGAAGTTGGGGCGGGGGGTCAAAAAGTCAAAACAACGAAAAGTAGTGGAGACGCAGGTAGTTAGACACAACTTGTTACACTTTTATTGTGTTAGTTTGAATCAAAAAATCACTAAATTTCAAGTTTTCTGAAATAATTATTTCTTATTTTTTGAAAAAAAATCTGAAAATTTTGATTTTTTCAGGCCAAAATTTTGAGCAATGAAGCTTCAAATTTTCTACTCAATTTTATTGATTTTTGTGGAATTTTTGGTGGATCCGAGTTCAAGGTGAGCGTTCAAACTTATTTATTGATTTTTTTCAAAATTCAAAAAATAAAAAAATTAATTTTTTTTTCGAATTTTCAAAAAAAAATTAAAATTTTTTCTCCAAAAAAATTTCGTTCTCACTCAATTTTTCTCAACTTTTTCGAATTAACAGTCAAAAAAACCAAATTTTCAGCTTATTTTCCTCTACAAATCGAAAATTTGACCCTAATCGCCCGACAATGTCAAATCAGGGCCATCGGATGGGGTGGCCAGTACAGCAGAGAAAAGCCAATGAGGAAGTGGTGAATAGCCAGAGAAACACCGAGTTTTTGATAATTCATTGAGAAAAATCAATAATTTTTGAGATATTAATAAAGGTTTTCACAATTCCAAGTTAAAAATCGAAGTTTGACCACTATTTTCGAGTTTCTTCTTGATTTTCTCCATTTTTAAGAAAAAAATTCGCAATTTACATAGTGGAAAACCTAAATTCAACCTTGGGGCCTCGAACTTACCTCATTTTTTTCTTTTCGATCCATGGCCAAGGCTGTTACCGGCTGCGCCACGCGCGCGCTGGCCCCGCACTCTAGTTTTCTCTGCCCGCGTGGCGCAGTGGGTAACAGAGATGCCTTCGGATCTGAAATGAGAAAAAGGGGGGCAAGTTCGAGCCCCCAAGTGGGTTTTTAGGTTTTCTAATATGGGGATTCCGATTTTTCAGTTTAAAAATAACGAAAACCAAGTTGAAAACTACTTTTCTCACAGTTTTCCTTTTTTTTCCGGTTTTTTTGGATATTCAAGCACGTAATGTTCCCATTCAGGTGAACTACGTCTTAAATCTAATAATCAACTTCCTGACGTCCGAGTTCCGGGTACTTATGCTGCTATTAATGCACACGCGGAAAGGGGTTCAAATTGCCGGAACCACCGGATTATCCTTGTGTTTTTTTTTCGTTTGTTATTGAATACATTTTTTGCTTATTTTTTATCTAAAAATAAAATTCTTAAAAATCTTGAAATAAAAAAAAAATCCAAAAAATTTTTGACATTTTTTTTTCAAAATTTTCAAAAAAAAAAAAAAACCAGCCCATTTTAAATATATATTTTAAGTACTCAAAATTATTCGAAACTGATCTACTAACTTAAAACTTCCAAAAAAAATCAACTCCGAAAGTATCAAAAATCGCGAAAATTTTTGCTCGCGGAGCCCGTGGAGCAATAAAACTCAAAAAACATTCAAAATCATCGCGGCAATGTTTTTATTAATCTATAACTCTTCCATTTCTCCCCCGTTCTTCTCAATTTCGCCCATTTTTTCCCCGTTTTTATGTTCCAGTGAGTGAGCTTCCATGATGTTCCCATGGGACCTCCCTTGATTCTATAGATTTCTCTTTATCTTTTTGCATCTTTTTCTCCTATTTTTCACATTTTTCAGGCATTTTTTCAAAATGAACGTTGAAAGGTTTTTAGTTTAAATTGAAAAAATTAAAAAAAAAATTTTGAATTTTTTTGGAATTTTTGGGTCTCAGCAAGAAAATTTTTTGATTACTGTAGTTATTGGAAATTAATTTTTTAAGTCAAAAATTGGTACTTTTCACTTGCCCTCTTCACCAGAAAAGCTCTAGAAAAAGCTCGGCTTCAGTTTTTTTCCCAAAAATCTTTTTTTTCTTCCAAATTTCGTATAAATTCATAACCCGTCACTTTTGACTTTCCTCATTCGTTTCACAAAACGTCAATTCATTTTAGTGTAGACGGGGCCGGCGGGTGTCCCCAAAAAAAGGTAATGCATTTTTGATTTTCTTTGGGGGCCCTGAGATAACACACTTTTTTATTATTAGAGAAAAAAACACTTTTTTATTAATTTATTTTTTCTTGAAAATCTAGTTTTCATGTTGATTTCACCAAATTTTAAGTCAAAAATTTGAAATCACCACTATCGAAAACCTAAATCCCTATTCAGGAC
