# Supplementary material for: Identification of functional long non-coding RNAs in C. elegans
Source: BMC Biol. 2019 Feb 18;17:14. doi: 10.1186/s12915-019-0635-7 (PMC6378714; doi:10.1186/s12915-019-0635-7)
Supplement: Supplementary file 10 — Extended methods for automated microscopy and phenotyping (PDF 197 kb) [file 12915_2019_635_MOESM10_ESM.pdf]

1 Supplemental Methods: Phenotypic Analysis

Synchronization by coordinated egg laying To generate populations of synchronized animals without bleaching and starvation, young egg-laying adults (50-75 hours post hatching) were gently picked with a platinum wire to a fresh NGM plate seeded with the appropriate bacteria and allowed to lay eggs for a fixed duration, after which the adults were removed from the plate and the eggs were collected. The egg-laying rate of animals at this stage is ≈ 6 eggs/animal/hour. Synchronization could be tightened by shortening the duration of egg-laying, and the number of synchronized eggs could be increased by using more egg-laying adults.

For these data, 10 adults were used and synchronization was to within one hour resulting in populations of ≈ 50 synchronized individuals.

Parallel imaging system specifications The imaging system consists of a custom built PC which records videos from 12 Point Grey Flea3 cameras (FLIR Systems, Wilsonville OR, USA) in parallel. The system was designed to have sufficient USB 3.0 bandwidth and PCI-e lanes for the 12 cameras to operate simultaneously. Cameras were fitted with a 25 mm EFL, f/1.4 lens offset with a 10 mm C-mount extension tube (Thorlabs, Newton NJ, USA)(See Table 1). This set-up gave an effective magnification of 0.22x, resulting in $11.3 \mu m/pixel$. Videos were recorded at 1 Hz, in MPEG format with a quality of 0.75, using the FlyCapture software provided with the cameras and stored to hard disk drives for off-line processing.

Table 1: Parallel Imaging System Components

**PC**	
Motherboard	ASUS X99-Deluxe
Processor	Intel Core i7 6700K 4.0 GHz
USB 3.0 Card	3x Startech PEXUSB3S44V
**Optics**	
Camera	Flea3 3.2 MP Mono - FL3-U3-32S2M-CS
Lens	25 mm EFL, f/1.4 w/ 10mm Extension Tube

Image Acquisition and Analysis Images were recorded as MPEG compressed videos at a compression quality of 75% using the Point Grey Fly Capture software. Compression at this level had no measurable effect on

the size and spacings of measurements from a standard test target (Edmund Optics, Barrington NJ, USA). Frames from these movies were extracted into MATLAB (Mathworks, Natick MA, USA) using the VideoIO toolbox (Gerald Dalley). Moving objects were extracted from each image using background subtraction. The background image was recalculated every ≈ 17 minutes to account for slow changes in background illumination. Background subtracted images were thresholded, and the locations and properties of connected components (”blobs”) were recorded from the resulting black and white image. Area (in pixels), and centroid location were calculated using the MATLAB function *regionprops*, and length was computed using the MATLAB functions *bwmorph* to extract the skeleton, the function *bwgeodesic* to compute the length.

Nematode Growth Media Animals were maintained on Nematode Growth Media (NGM) plates. The recipe is given in Table 2. For experiments done with NGM (-)Peptone plates, the recipe was identical except that the Peptone was omitted. NGM (-)Peptone agar was used to prevent bacterial growth on the plate, so that the only available bacterial food was that which was initially inoculated. Additive salts were prepared in 1 M stock solutions, and the KH_2PO_4 stock solution was adjusted to pH 6. Cholesterol was prepared as a 5mg/ml stock solution in ethanol.

Table 2: Nematode Growth Media Plates

Ingredient	Amount
Autoclave Together	
NaCl	3.0 g/l
Agar	17.0 g/l
Bacto-Peptone	2.5 g/l
Add Aseptically	
$CaCl_2$	1 mM
$MgCl_2$	1 mM
Cholesterol	5mg/l
KH_2PO_4	25 mM

Mini-well fabrication Mini wells were made by gluing with cyanoacrylate a pre-cut 5mm acrylic donut into a standard 30mm Falcon petri dish.

The donut forms were cut from the acrylic using a LS 6090 PRO Laser Cutter (HPC Laser Ltd, Halifax, UK). The resulting mini-well was $16mm$ in diameter and had a volume of $1ml$. This well was filled with NGM-Agar (-)Peptone media, allowed to set and dry for 1 hour covered at 20C, and seeded with $20\mu l$ of bacteria at a optical density of 10 OD, corresponding to approximately 180 million cells, as measured using a Petroff-Hausser Counting Chamber (Hausser Scientific, Horsham PA, USA).

Temperature Control Plates were kept in an insulated, temperature controlled box during imaging, which itself was in a temperature controlled room set to 20C. The actual average temperature in the room was 19.7C. Temperatures were measured with a custom thermometer; the signal from a linear thermistor (Omega Engineering, Stamford CT) was difference amplified against a known voltage corresponding to 20C. The amplified signal (Gain = 2) was recorded in MATLAB (Mathworks, Natick MA) from the analog input of a DAQ (Labjack, Lakewood CO). This was fed into a Proportional Integral Differential (PID) control script whose output was a voltage that controlled a Push-Pull current amplifier driving a Peltier effect element (Custom Thermoelectric, Bishopville MD, USA). Fans were used to distribute air from the heat sinks on each face of the Peltier element either into the enclosure or as exhaust. Temperature was maintained to within 70 mK of the set point (20C).

Automated Counting The number of worms on a plate was counted automatically using a single 8.8 MP Point Grey Flea3 camera. This was done by first tapping the plate gently 5 times to induce the worms to move. After this, custom MATLAB software recorded images at 1Hz for 15 seconds, and a background image was calculated as the pixel maximum over all images. Subsequently, for 15 additional frames, worms were detected by thresholding background subtracted images for intensity, and then by filtering out non-worm blobs using a series of size and intensity filters. The number of worm objects in each frame was stored and the median value over the 15 frames was used as the count. Counts produced in this way had a precision of 3% over repeated measurements of the same plate, and the median value had an accuracy of 5% for measurements of plates with a known number of worms..

Comparison of automated microscopy to manual counting

We compared the automated microscopy accuracy to the accuracy of 7 individuals accustomed to manually counting *C. elegans* larvae on agar plates using stereo microscopes.

at low densities (10 animals) both human and automated counting perfectly accurate. At high density (99 animals), the human measurements (n=3 counts for each of N=7 individuals), the highest human accuracy was 1.7% and the average was 4.2%, with a precision of 3.9%. The machine accuracy was 2% with a precision of 2.8%.

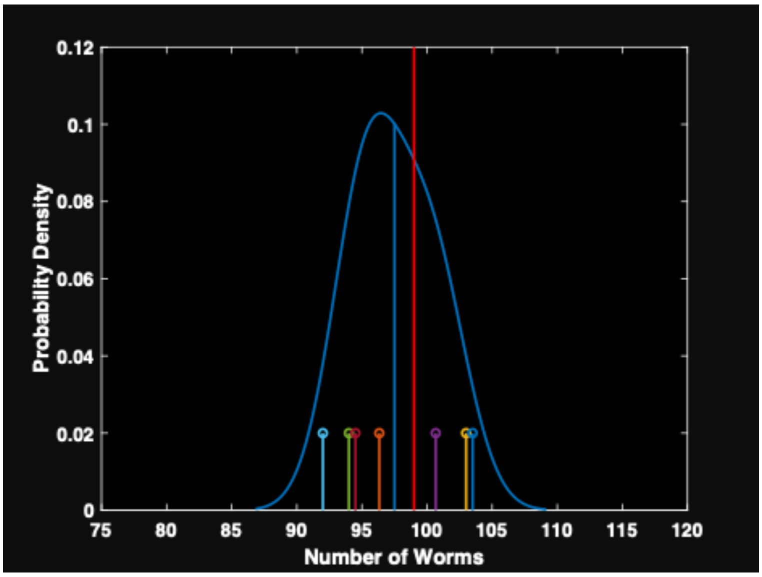

Figure A. Probability Densities for automated counting compared to mean individual manual counts. For a true number of worms (99), N=7 individuals counted each plate n=3 times. The stem plot gives the average of these three measurements for each individual. The blue distribution gives a kernel density estimate of the probability distribution of the automated counts (n=21), with its median given as the blue vertical line. On average, the machine missed the true number by 2%. The best human taking 3 measurements was slightly more accurate at 1.7%.

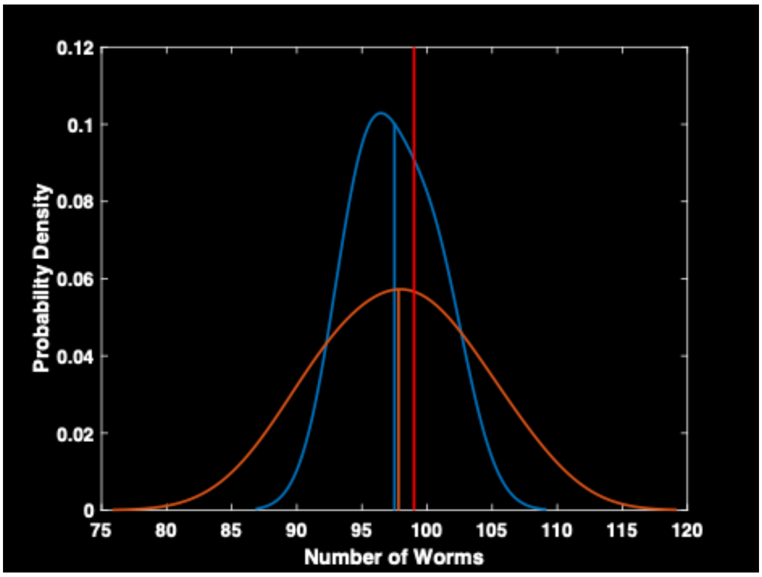

Figure B. Probability Densities for manual and automated counting measurements. For a true number of worms (99), the best estimate of the probability distribution of all of the measurements (n=21) for the automated microscope and for the collected human measurements are shown as calculated by a kernel density estimator. The average accuracies of the two methods are comparable, but the precision of the automated measurement is greater.
